# Microglia pre-activation and neurodegeneration precipitate neuroinflammation without exacerbating tissue injury in experimental autoimmune encephalomyelitis

**DOI:** 10.1186/s40478-019-0667-9

**Published:** 2019-01-31

**Authors:** Isabella Wimmer, Cornelia Scharler, Tobias Zrzavy, Taro Kadowaki, Verena Mödlagl, Kim Rojc, Anna R. Tröscher, Maja Kitic, Shuichi Ueda, Monika Bradl, Hans Lassmann

**Affiliations:** 1 Department of Neuroimmunology, Center for Brain Research, Medical University of Vienna, Spitalgasse 4, 1090 Vienna, Austria; 2 Department of Neurology, Dokkyo Medical University, Tochigi, Japan; 3 Department of Histology and Neurobiology, Dokkyo Medical University, Tochigi, Japan

**Keywords:** Progressive multiple sclerosis, Experimental autoimmune encephalitis, Neurodegeneration, Microglia, Zitter rat

## Abstract

Human inflammatory or neurodegenerative diseases, such as progressive multiple sclerosis (MS), occur on a background of age-related microglia activation and iron accumulation as well as pre-existing neurodegeneration. Most experimental models for CNS diseases, however, are induced in rodents, which are naturally characterized by a homeostatic microglia phenotype, low cellular iron load and absence of neurodegeneration. Here, we show that naïve LEWzizi rats – Lewis rats with a zitter rat background – show a spontaneous phenotype partly mimicking the changes seen in human aging and particularly in the normal-appearing white and grey matter of patients with progressive MS. Using this model system, we further aimed to investigate (i) whether the acute monophasic MS model experimental autoimmune encephalomyelitis (EAE) transforms into chronic progressive disease and (ii) whether EAE-induced neuroinflammation and tissue damage aggravate on the LEWzizi background. We found that the pre-existing LEWzizi-specific pathology precipitated EAE-related neuroinflammation into forebrain areas, which are devoid of EAE lesions in normal Lewis rats. However, EAE-related tissue damage was neither modified by the LEWzizi-specific pathology nor did EAE-induced neuroinflammation modify the LEWzizi-related pathological process. Our data indicate that the interaction between pre-activated microglia and CD4^+^ autoreactive T cells during the induction and propagation of tissue damage in the CNS is limited.

**Electronic supplementary material:**

The online version of this article (10.1186/s40478-019-0667-9) contains supplementary material, which is available to authorized users.

## Introduction

Multiple sclerosis (MS) is a chronic neuroinflammatory disease, which in the majority of patients starts with a relapsing/remitting disease course [32]. In this stage of the disease, new focal lesions are formed mainly within the white matter and are characterized by an influx of T and B cells into the brain associated with blood-brain barrier damage, primary demyelination and reactive astrocytic scarring [25]. After ten to fifteen years, the relapsing/remitting disease course converts into a phase of steady progression (secondary progressive MS). Some patients skip the relapsing disease stage and already start with steady progression (primary progressive MS) [32]. The pathological substrate of progressive MS is the slow expansion of pre-existing lesions in the white matter and the cerebral cortex as well as diffuse neurodegeneration in the entire normal-appearing white and grey matter [26]. While the pathology of relapsing/remitting MS is partially reproduced in models of experimental autoimmune encephalomyelitis (EAE), no experimental animal model has yet been described that reflects the pathological changes in the progressive stage of MS [24]. Recent studies on autopsy tissue from patients with progressive MS suggest that a cascade of events, including microglia activation, oxidative injury, mitochondrial damage and the resulting energy deficiency (virtual or histotoxic hypoxia), plays a major role in driving demyelination and neurodegeneration in the progressive stage of the disease [8, 9, 34].

Since progressive MS develops in an age-dependent manner [46, 60], it has been suggested that age-related changes in the brain contribute to the propagation of the neurodegenerative process in the affected patients. In humans, brain aging is associated with chronic microglia activation [39], iron accumulation in myelin and oligodendrocytes [12], a pro-oxidative state of the brain [45], mitochondrial alterations as well as progressive myelin damage and neurodegeneration [51]. Thus, inflammatory MS lesions in aging patients develop on the background of a pre-injured CNS, which is not reflected in rodent EAE models. We therefore hypothesized that neuroinflammation induced on a background of pre-existing inflammatory and neurodegenerative conditions may be amplified and even convert from an acute self-limiting monophasic disease into chronic progressive disease like in the progressive stage of MS.

To test this hypothesis, we made use of a rat model (LEWzizi) that originally descended from the zitter (zi/zi) rat, a spontaneous attractin (*Atrn*) mutant [22, 42] presenting with a variety of neuropathological features that resemble key aspects of MS pathology, such as neurodegeneration [52, 53], hypomyelination [18], microgliosis [16, 49], extensive iron accumulation [49] and dysregulated anti-oxidative systems [11]. Here, we first characterized the newly established LEWzizi rat model and further investigated whether pre-existing microglia activation, hypomyelination and axonal damage amplify neuroinflammation and tissue injury in passive MBP-EAE. We observed a minor increase of clinical disease; however, the combination of a pre-injured CNS environment with the induction of EAE did not lead to an exacerbation of oligodendrocyte or axonal pathology. Interestingly, we observed a topographic shift of inflammation spreading from the spinal cord to forebrain regions, which were pronouncedly pre-affected by zitter pathology.

## Materials and methods

### Animals

For immunological purposes, zitter (zi/zi) rats (Sprague-Dawley outbreds) were backcrossed for three generations on the Lewis background resulting in LEW.SD-*Atrn*^*zi/zi*^ rats denoted as LEWzizi rats throughout this publication. Lewis and LEWzizi rats were housed in the Institute for Biomedical Research (Medical University Vienna) under standardized conditions. All experiments were approved by the Ethic Commission of the Medical University Vienna and performed with the license of the Austrian Ministry for Science and Research.

### Tissue sampling

Rats were routinely killed by an overdose of CO_2_ and perfused intracardially either with 4% paraformaldehyde (PFA) or phosphate-buffered saline (PBS). For histological analysis, brain and spinal cord were dissected, post-fixed in 4% PFA for 24 h and routinely embedded in paraffin. For gene expression analyses, lumbar spinal cord was dissected, snap-frozen and stored at − 20 °C or − 80 °C until RNA isolation.

### Histological staining procedures

3,3′-diaminobenzidine tetrahydrochloride hydrate (DAB)-developed immunohistochemical single stainings were routinely performed on deparaffinized and rehydrated formalin-fixed paraffin-embedded FFPE tissue sections [3]. Antigen retrieval for immunohistochemistry (Table 1) was performed for 1 h by steaming of the tissue sections in either 10 mM citrate buffer (pH 6.0) or 1 mM EDTA in 10 mM Tris buffer (pH 8.6). Primary antibodies (Table 1) were incubated overnight at 4 °C. Biotinylated secondary antibodies and peroxidase-conjugated streptavidin were each applied for 1 h at room temperature (RT). Selected primary antibodies (Table 1) required a catalysed signal amplification (CSA) step after the peroxidase-conjugated streptavidin incubation. For this, tissue sections were incubated with biotinylated tyramine [3, 17] for 20 min and once more with peroxidase-conjugated streptavidin for 30 min. Thereafter, DAB development was done.

**Table 1:** Primary antibodies and modes of antigen retrieval for immunohistochemistry

Antibody	Origin	Dilution	Antigen retrieval	CSA	Source
Amyloid precursor protein (APP) [clone 22C11]	Mouse (mAb)	1:1000	St (C)		Chemicon, Merck Millipore; MAB348
CD3 [clone SP7]	Rabbit (mAb)	1:2000	St (E)	yes	Neomarkers; RM-9107
CD68 [clone ED1]	Mouse	1:10,000	St (E)		Serotec
2′,3’-Cyclic-nucleotide 3′-phosphodiesterase (CNPase) [clone SMI 91]	Rabbit (mAb)	1:2000	St (E)		Sternberger Monoclonals; SMI91
Glial fibrillary acidic protein (GFAP)	Rabbit (pAb)	1:3000	St (E)		Dako; Z0334
Ionized calcium-binding adapter molecule 1 (Iba-1)	Rabbit (pAb)	1:3000	St (E)		Wako Chemicals; 019–19741
Inducible nitric oxide synthase (iNOS)	Rabbit (pAb)	1:750	St (C)		Chemicon, Merck Millipore; AB16311
MHC class II RT1B [clone OX-6]	Mouse (mAb)	1:250	St (E)		Serotec; MCA46G
Neuronal nuclei (NeuN) [clone A60]	Mouse (mAb)	1:250	St (C)		Chemicon, Merck Millipore; MAB377
Chondroitin sulfate proteoglycan 4 (NG2)	Rabbit (pAb)	1:250	St (E)		Chemicon, Merck Millipore; AB5320
Oligodendrocyte transcription factor 2 (Olig2)	Rabbit (pAb)	1:1000	St (E)		Chemicon, Merck Millipore; AB9610
p22phox [clone FL-195]	Rabbit (pAb)	1:100 for pre-absorption^a^	St (C)		Santa Cruz Biotechnology; sc20781
Purinergic receptor P2Y12 (P2RY12)	Rabbit (pAb)	1:1000	St (E)	yes	Kindly provided by Oleg Butovsky (Harvard Medical School)
Transmembrane protein 119 (TMEM119)	Rabbit (pAb)	1:1000	St (E)		Synaptic Systems; 400002
ATP synthase alpha [clone 7H10]	Mouse (mAb)	1:200	St (E)		Thermo Fisher Scientific; 459240
Phosphorylated neurofilament H [clone SMI 31]	Mouse (mAb)	1:20,000	St (E)		Affiniti; NA1219

St, steaming of sections using the indicated buffer solution; C, 10 mM citrate buffer (pH 6.0); E, 1 mM EDTA in 10 mM Tris (pH 8.6); mAb, monoclonal antibody; pAb, polyclonal antibody; CSA, catalysed signal amplification via biotinylated tyramine enhancement

^a^p22phox antibody was pre-absorbed with Lewis cortex homogenate in order to reduce unspecific background staining

DAB-enhanced Turnbull’s blue (TBB) staining for the detection of non-haem iron was done as described [13, 35]. For double labelling of non-haem iron with Olig2, the TBB staining was developed with DAB for 2 h; for double labelling with Iba-1, with AEC for up to 2 h. Thereafter, antigen retrieval (Table 1) was done for 45 min and after a blocking step, primary antibodies (Table 1) were incubated overnight at 4 °C. Afterwards, alkaline phosphatase-conjugated secondary antibodies were applied and antibody labelling was developed with Fast Blue [3].

### Histological analysis

Cell counts and numbers of APP-stained neuronal spheroids and endbulbs within regions of interest (ROIs) were manually determined using standardized counting grids within the microscope oculars. Generally, counting was performed with a 20 × objective lens with the exception of APP and CNPase stainings, for which a 40 × objective lens was chosen. For the analysis of spinal cords, 2 to 6 counting grids were fit into each ROI; two lumbar cross sections per rat were analysed. For the analysis of coronal brain sections, 1 to 4 counting grids per hemisphere were fit into each ROI. To determine the degree of myelination, pictures (2 lumbar sections analysed per rat and 2 pictures taken per spinal cord cross section; 2 pictures taken per coronal brain section) were taken with a 25 × objective within the respective ROIs of CNPase-stained tissue sections and the integrated density of the antibody labelling was measured in ImageJ (v1.48). To analyse the expression of inflammatory markers during EAE, pictures of lumbar spinal cord cross-sections were analysed in ImageJ as well. To distinguish between DAB staining and background noise, a uniform threshold was applied across each staining type and the area fraction, representing the positively stained area per picture, was measured. For representative purposes, slides were scanned at a NanoZoomer 2.0HT employing the NanoZoomer Digital Pathology scan software v2.5.85 (Hamamatsu Photonics K.K.).

### T cell culture

Lewis and LEWzizi rats (male; 3-month-old) were immunised subcutaneously into the tail root with 200 μl of a mixture of (i) 20 μg myelin basic protein (MBP; Sigma-Aldrich, M2295) diluted in RPMI medium to a final volume of 100 μl and (ii) 100 μl complete Freund’s adjuvant (CFA) containing 400 μg *Mycobacterium tuberculosis* H37 Ra (DIFCO, 231141 and 263,910). Ten days post immunization, animals were killed by an overdose of CO_2_, the draining peri-aortal lymph nodes were harvested and MBP-specific T cell lines were established and re-stimulated for several rounds as published [5]. For the induction of passive EAE, activated T cell blasts 24 h post re-stimulation were used.

### Passive EAE

Lewis and LEWzizi rats (4- or 8-month-old; male and female) were injected intraperitoneally with Lewis- or LEWzizi-derived MBP-specific T cell blasts resulting in the following combinations per age group: Lewis rat injected with Lewis T cells, Lewis rat injected with LEWzizi T cells, LEWzizi rat injected with Lewis T cells and LEWzizi rat injected with LEWzizi T cells. Animals were weighed daily and clinical symptoms of EAE were scored according to the following rules on a daily basis: 0, healthy; 1, complete loss of tail tonus; 2, partial hind limb paresis (unsteady gait); 3, complete hind limb paralysis. If the latter criterion was met, animals were sacrificed instantly for ethical reasons. Otherwise, CNS tissues were routinely harvested either 6 days (peak of the disease) or 10 days (recovery phase) post disease induction.

### RNA isolation

RNA was isolated from 40 mg tissue using the peqGOLD Total RNA Kit and peqGOLD DNase I Digest Kit (both Peqlab, VWR). RNA quality was routinely determined via Agilent RNA Nano chips using the 2100 Bioanalyzer (Agilent Technologies) and stored at − 80 °C until further processing.

### Whole-genome microarrays

60 ng total RNA were used for cDNA preparation using the GeneChip™ WT Plus Kit (Thermo Fisher Scientific) according to the manufacturer’s instructions. 3.5 μg of labelled and fragmented samples were hybridized to Affymetrix GeneChip™ Rat Gene 2.0 ST Arrays (Thermo Fisher Scientific) and microarrays were scanned on a GeneChip™ Scanner 3000 7G (Thermo Fisher Scientific). The resulting CEL files were loaded in the Transcriptome Analysis Console software (v4.0.1.36), normalized via Robust MultiArray Average (RMA) algorithm and evaluated via eBayes-corrected analysis of variance (ANOVA) and Benjamini-Hochberg false discovery rate (FDR)-adjusted *p*-values. Differentially expressed genes (± 1.2 fold-change; FDR-corrected *p*-value ≤0.05) were subjected to GO-term-based enrichment analysis via a free online tool (http://geneontology.org/page/go-enrichment-analysis) using PANTHER overrepresentation test (release 20171205) and either Reactome version 58 (release 2016-12-07) or GO Ontology database (release 2018-05-21) for pathway annotation. Statistical testing based on Fisher’s exact test with FDR multiple test correction. For graphical data representation, z-scores were calculated for each gene of interest. Microarray data were deposited in NCBI’s Gene Expression Omnibus repository (GSE119793).

### Statistics

For statistical analysis and data representation, GraphPad Prism® v6.01 was used. To determine differences between naïve Lewis and LEWzizi rats, unpaired two-tailed Student’s t-tests were calculated. For EAE experiments, putative effects of the two independent variables “rat genotype” and “T cell genotype” on the investigated clinical parameters and immunohistochemical data were tested via two-way ANOVAs and results are presented in Additional file 1: Table S1. Since these results indicated a negligible influence of the T cell genotype, data were subsequently pooled according to rat genotype and independent of T cell genotype. For further statistical testing, two-way ANOVAs combined with Sidak’s multiple comparisons tests using naïve control data sets and EAE data sets were calculated (separate analyses for day 6 and day 10). Generally, a *p*-value < 0.05 was considered statistically significant.

## Results

Since we intended to induce EAE in the zitter rat model, we first had to transfer it to an immunologically compatible background. For this, we crossed zitter rats (Sprague-Dawley background) with wild-type Lewis animals resulting in LEWzizi (LEW.SD-*Atrn*^*zi/zi*^) rats. They exhibited the same phenotypic traits as described for the original zitter model [42] such as moderate generalized body tremor, unsteady gait and age-dependent moderate progressive flaccid paresis of the hind limbs (data not shown).

### Microglia pre-activation, abnormal iron accumulation, chronic myelin pathology and axonal injury in the CNS of LEWzizi rats

**Fig. 1:**
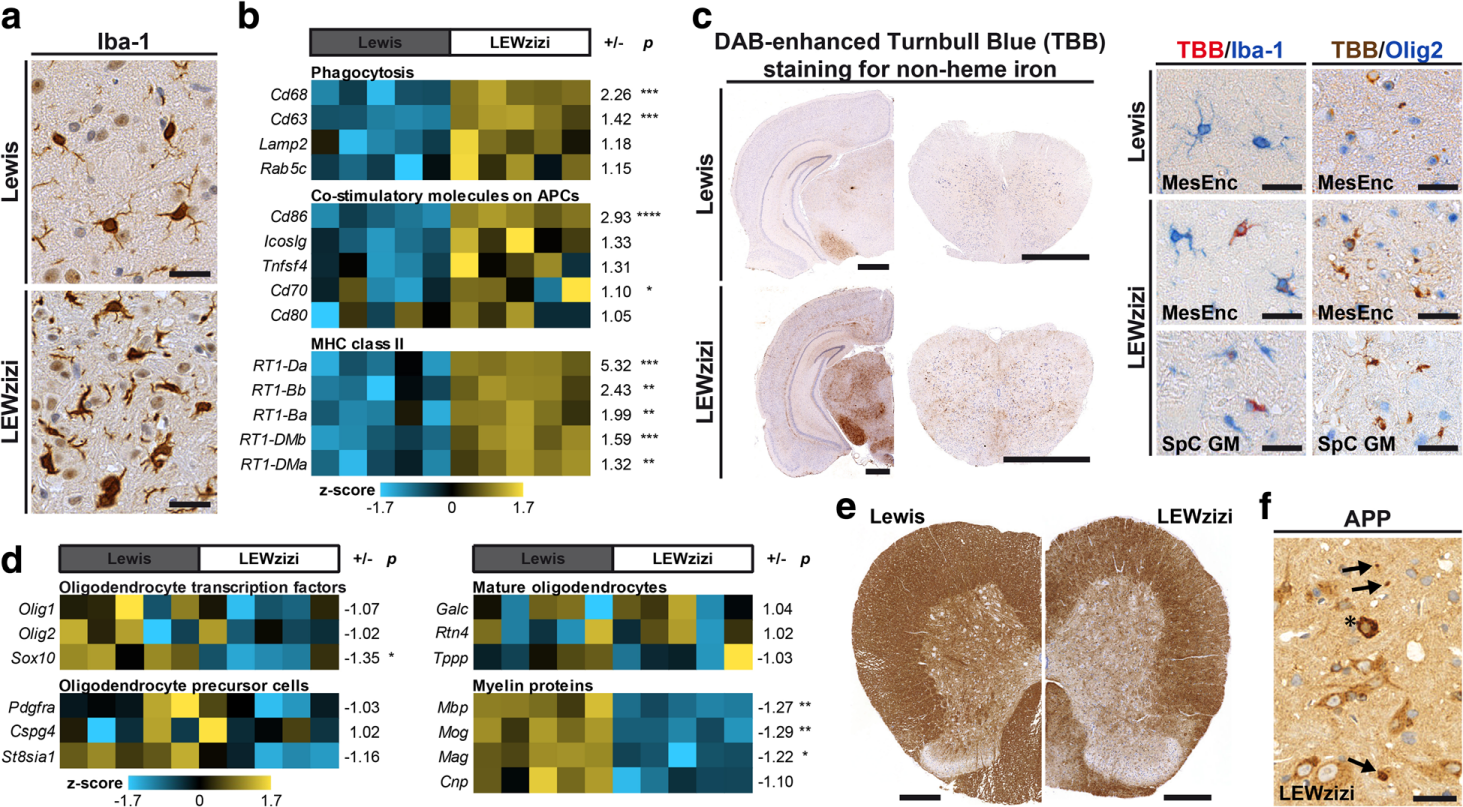
Microgliosis, abnormal iron accumulation, oligodendrocyte/myelin pathology and axonal injury in the LEWzizi CNS. **a** Immunohistochemical staining for Iba-1 of lumbar spinal cord grey matter (SpC GM) of 4-month-old (4 M) Lewis and LEWzizi rats. Scale bars, 25 μm **b** Gene expression analysis of microglia-associated genes in lumbar spinal cord homogenates of 4 M rats. Per gene of interest, color-coded z-scores for each biological replicate (*n* = 5 per rat strain) are shown as well as the level of up- or downregulation and the false discovery rate (FDR)-corrected *p*-value in LEWzizi compared with wild-type Lewis tissue. **c** Turnbull Blue (TBB) staining for the detection of non-haem tissue iron. Representative pictures of DAB-enhanced TBB single labelling as well as double stainings of AEC- or DAB-enhanced TBB with cell type-specific markers Iba-1 (microglia) or Olig2 (oligodendrocytes), respectively, derive from coronal CNS sections of 8-month-old (8 M) Lewis and LEWzizi rats. MesEnc, mesencephalon; scale bars, either 1 mm or 25 μm **d** Gene expression analysis of oligodendrocyte and myelin genes in lumbar spinal cord homogenates of 4 M rats. Per gene of interest, color-coded z-scores for each biological replicate (*n* = 5 per rat strain) are shown as well as the level of up- or downregulation and the false discovery rate (FDR)-corrected p-value in LEWzizi compared with wild-type Lewis tissue. **e** Immunohistochemical staining for CNPase of SpC GM cross sections of 4 M Lewis and LEWzizi rats. Scale bars, 250 μm. **f** Immunohistochemical staining for APP of a SpC GM cross section of an 8 M LEWzizi rat. APP-positive axonal spheroids and endbulbs are indicated by arrows; an oligodendrocyte exhibiting a strong cytoplasmic APP staining is indicated by an asterisk. Scale bar, 25 μm

Similarly as it has been previously reported for zitter rat brains [16, 18–20, 37, 49], we observed pronounced microgliosis, demyelination and neurodegeneration in the entire CNS of LEWzizi rats. The most severely affected areas were the spinal cord grey matter and the mesencephalon, on which we focused for further quantitative analyses. Numbers of microglia were increased up to 4-fold in the LEWzizi CNS (Additional file 1: Figure S1a) and they appeared activated and had shorter and thicker cell protrusions; yet, they were still ramified (Fig. 1a). LEWzizi microglia expressed the microglia-specific marker TMEM119 (Additional file 1: Figure S1f) and a fraction of the cells still expressed the homeostatic microglia marker P2RY12 (Additional file 1: Figure S1b). As shown by immunohistochemistry of tissue sections and genetic profiling of lumbar spinal cord homogenates, LEWzizi microglia expressed various activation markers (Fig. 1b; Additional file 1: Figure S1 and S2, Additional file 1: Table S2). Next to this pronounced microgliosis, we also observed a significant increase in the numbers of glial fibrillary acidic protein (GFAP)-positive astrocytes in LEWzizi rats (Additional file 1: Figure S3a, b). Moreover, we noticed an abnormally increased, age-related iron accumulation in the LEWzizi CNS, which was particularly elevated in the deep grey matter nuclei and to lesser, varying extents in other brain and spinal cord regions (Fig. 1c; Additional file 1: Figure S3c). Iron accumulation in oligodendrocytes and dystrophic axons was primarily found in the brain, while iron positivity in microglia was observed in both brain and spinal cord (Fig. 1c).

Oligodendrocyte counts in the mesencephalon and spinal cord grey matter of LEWzizi rats were significantly reduced (Additional file 1: Figure S4a, b). This was associated with a significant reduction of myelin gene expression (Fig. 1d) and myelin protein density (Fig. 1e; Additional file 1: Figure S4c) as well as the presence of myelin granules (Additional file 1: Figure S4d), which oftentimes co-localised with Iba-1^+^ microglia (Additional file 1: Figure S4e). Individual oligodendrocytes in the LEWzizi CNS exhibited a strong, cytoplasmic reactivity for amyloid precursor protein (APP) (Fig. 1f; Additional file 1: Figure S4 g) indicating oligodendrocyte stress as described in myelin protein mutant animal models [2]. Axonal injury, determined by the accumulation of APP in neuronal spheroids and endbulbs (Fig. 1f), was constantly high in brain and spinal cord of LEWzizi rats within the investigated timeframe from 2 to 8 months (Additional file 1: Figure S4j). However, we observed neither a significant reduction of nerve cells bodies (Additional file 1: Figure S4i), nor differences in neuronal expression patterns of mitochondrial respiratory chain complex V (ATPase) and of phosphorylated neurofilament H (SMI31) (Additional file 1: Figure S4k, l).

Despite the progressive neurodegenerative phenotype in the LEWzizi animals, we did not detect any infiltration of peripheral immune cells into the CNS. Similarly to wild-type Lewis rats, CD3^+^ T cells were hardly present in the CNS parenchyma and the sparse cell counts did not differ between Lewis and LEWzizi rats in the brain or spinal cord (data not shown).

### Induction of neuroinflammation in the pre-injured LEWzizi CNS environment leads to an earlier disease onset

**Fig. 2:**
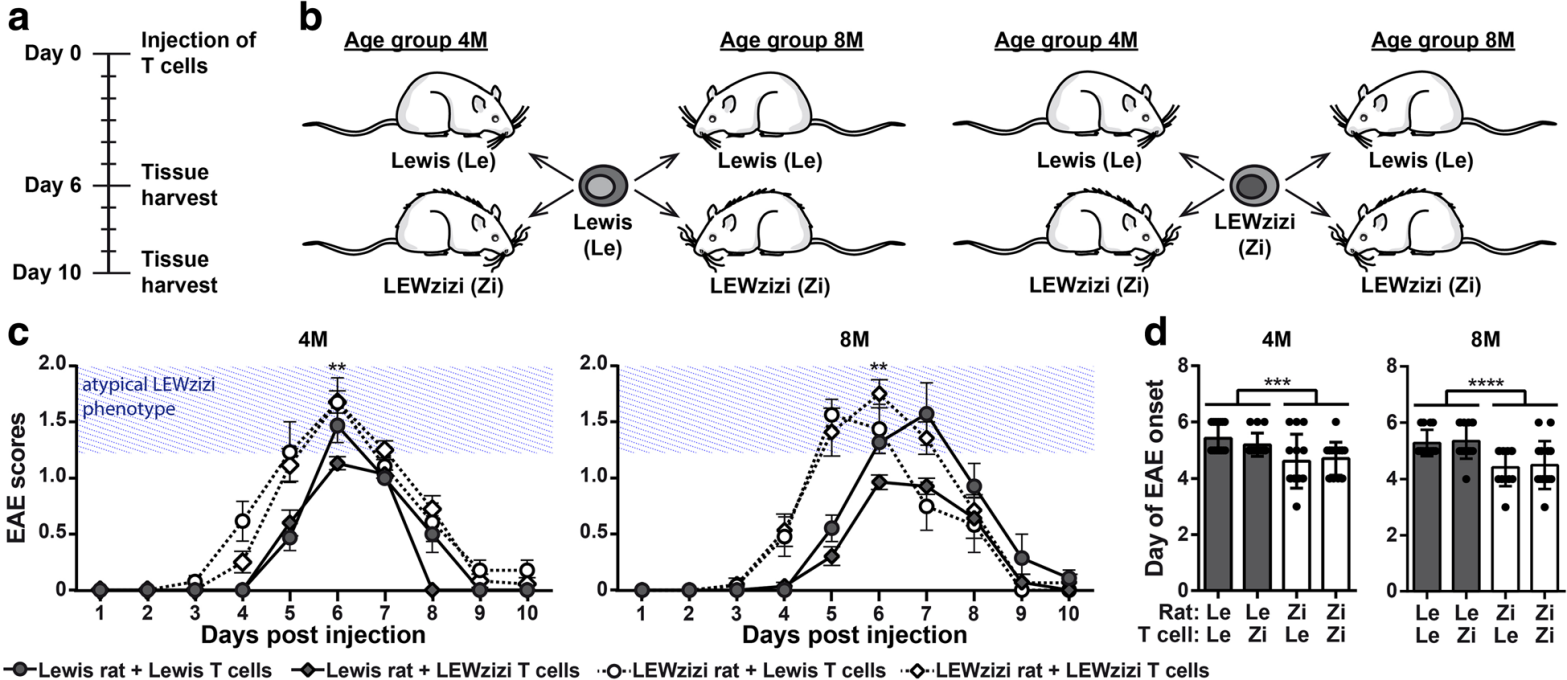
Clinical disease of passive MBP-EAE is more severe in LEWzizi rats. **a** Time axis of EAE experiments. **b** Injection scheme. MBP-specific activated CD4^+^ T cells originating from Lewis and LEWzizi rats were injected into naïve 4-month-old (4 M) or old 8-month-old (8 M) Lewis and LEWzizi recipient rats. **c** EAE disease course of 4 M and 8 M rats. Clinical signs of disease were scored on a daily basis as described in the Materials and Methods section. Blue shaded areas (“atypical LEWzizi phenotype”) indicate the score range that would overlap with LEWzizi-specific gait abnormalities. At the peak of disease (day 6), differences in disease scores between Lewis and LEWzizi rats (independent of the genotype of injected T cells) were tested via unpaired two-tailed Student’s t-tests. Graphs represent mean ± SEM. Each experimental group comprises 12–17 (day 1 to day 6) or 6–9 rats (day 7 to day 10). **, *p*-value < 0.01. **d** Start of clinical disease. EAE onset was defined as the first day of clinical signs > 0. Graphs represent mean ± SD. Each experimental group comprises 12–17 rats. Reported statistics result from unpaired two-tailed Student’s t-tests (data pooled according to rat genotype and independent of T cell genotype). ***, *p*-value < 0.001; ****, *p*-value < 0.0001

Our detailed analysis of LEWzizi pathology showed that this rat model is well suited to analyse the effects of pre-existing neurodegeneration, microglia activation and iron accumulation on experimentally induced neuroinflammation (using the EAE model) and, vice versa, the effect of EAE on neurodegenerative and inflammatory processes. To this end, we first established MBP-specific CD4^+^ T cell lines from immunised Lewis and LEWzizi rats. The cells showed overall comparable in vitro responses in antigen recognition and cell activation (data not shown). Subsequently, 4-months-old (4 M) and 8-months-old (8 M) naïve Lewis and LEWzizi rats were intraperitoneally injected with activated CD4^+^ effector T cells derived from Lewis or LEWzizi rats resulting in 4 experimental groups (Lewis rat + Lewis T cells; Lewis rat + LEWzizi T cells; LEWzizi rat + Lewis T cells; LEWzizi rat + LEWzizi T cells) per age group and day of tissue harvest (Fig. 2a, b). Both 4 M and 8 M LEWzizi rats consistently showed first signs of disease (loss of tail tonus) one day earlier than age-matched Lewis rats (Fig. 2d) and developed a more severe clinical disease course (Fig. 2c; atypical LEWzizi phenotype indicated by blue shaded areas). Statistical testing showed that clinical EAE was not influenced by the genotype of injected T cells (Additional file 1: Table S1). Our data suggest that the moderate increase in clinical disease in LEWzizi compared with Lewis rats resulted from EAE pathology adding up to LEWzizi symptomatology. Importantly, however, none of the animals developed a chronic progressive disease course.

### EAE-induced T cell recruitment to the pre-injured LEWzizi CNS differs in quantity and topography between brain and spinal cord

**Fig. 3:**
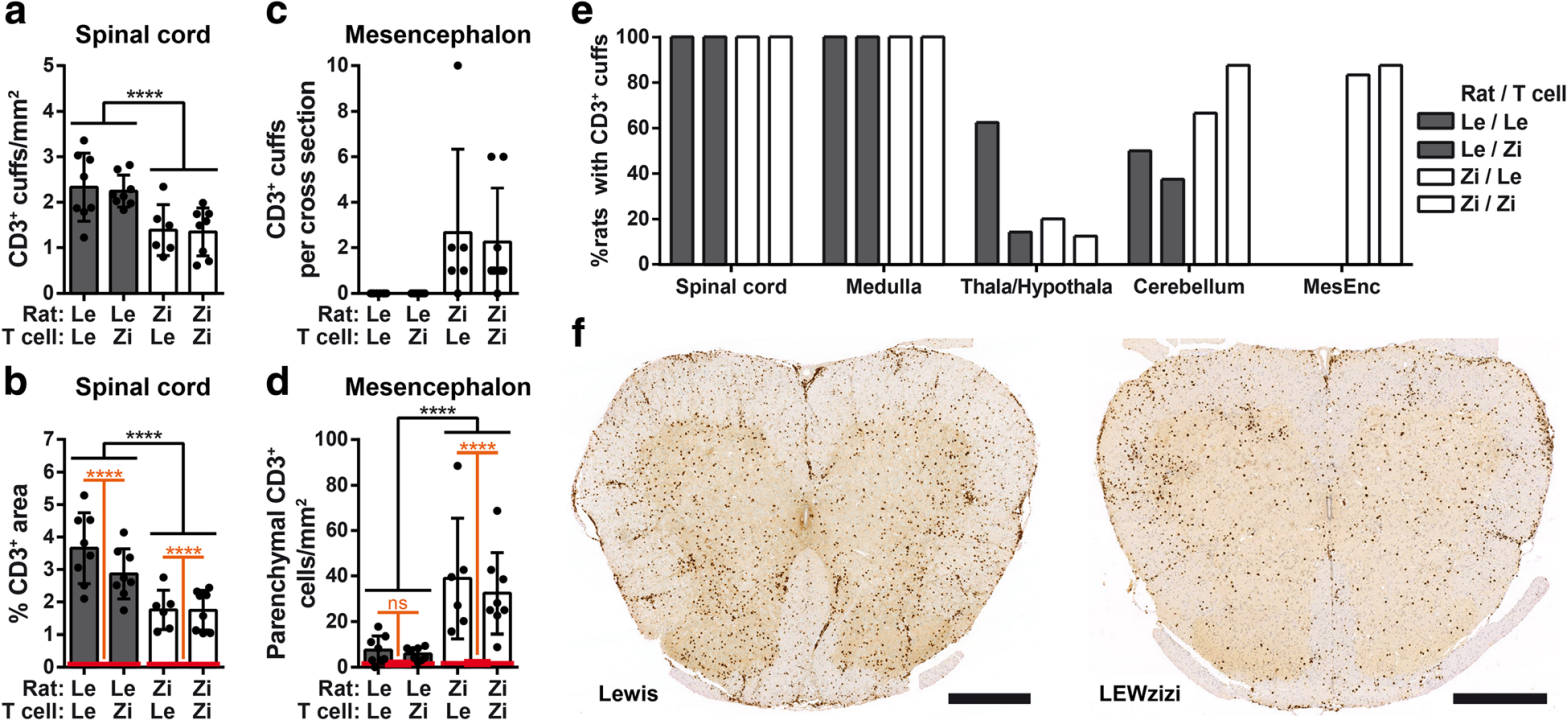
EAE-induced T cell infiltration differs between Lewis and LEWzizi rats. **a**, **c** Quantification of CD3^+^ T cell-containing perivascular cuffs in the lumbar spinal cord (**a**) and mesencephalon (**c**) of 4-month-old (4 M) Lewis and LEWzizi rats at the peak of EAE (6 days post transfer of MBP-specific T cells). **b** Area fraction analysis of CD3-stained lumbar spinal cord cross sections of 4 M Lewis and LEWzizi rats at the peak of EAE. The percentages of positively labelled area are depicted. **d** Quantification of parenchymal CD3^+^ T cells within the mesencephalon of 4 M Lewis and LEWzizi rats at the peak of EAE. **e** Evaluation of CD3^+^ inflammatory cuffs in the lumbar spinal cord, medulla oblongata, thalamus/hypothalamus (Thala/Hypothala), cerebellum and mesencephalon (MesEnc) of 4 M Lewis and LEWzizi rats at the peak of EAE. Experimental groups each comprise 6–8 rats. **f** CD3 immunohistochemical staining of lumbar spinal cord cross sections of 4 M Lewis and LEWzizi rats at the peak of EAE. Scale bars, 500 μm. **a**-**d** Graphs represent mean ± SD. Experimental groups each comprise 6–8 rats. ****, *p*-value < 0.0001; ns, not significant (**a**, **c**) Reported statistics result from unpaired two-tailed Student’s t-tests (data pooled according to rat genotype and independent of T cell genotype). **b**, **d** Red lines indicate the mean ± SD of age-matched naïve Lewis or LEWzizi controls. Statistics result from two-way ANOVAs (separate analyses for day 6 and day 10) reporting (i) differences between Lewis EAE rats and LEWzizi EAE rats by black bars and (ii) differences between naïve controls rats and EAE rats by orange bars. Data were pooled according to rat genotype and independent of T cell genotype

Contrary to clinical disease and despite pre-existing tissue injury, T cell infiltration in the spinal cord of LEWzizi rats upon induction of EAE was attenuated compared with diseased wild-type Lewis rats. The numbers of inflammatory cuffs with perivascular T cell accumulation at the peak of EAE were significantly lower in both 4 M and 8 M LEWzizi rats (Fig. 3a, f; Additional file 1: Figure S5a). Moreover, total CD3 immunoreactivity of spinal cord cross sections was strongly decreased for LEWzizi rats on day 6 post T cell transfer (Fig. 3b, f; Additional file 1: Figure S5c). Remarkably, the opposite pattern was seen in the mesencephalon. In this region, inflammatory infiltrates were largely absent in Lewis rats with MBP-EAE, while prominent inflammation was seen in LEWzizi rats with MBP-EAE (Fig. 3c, e; Additional file 1: Figure S5b). Similarly, parenchymal T cell numbers were significantly increased in the LEWzizi mesencephalon (Fig. 3d; Additional file 1: Fig. S5d).

These data indicate that the EAE-induced inflammatory process, which is largely restricted to the spinal cord in Lewis rats, might be topographically re-directed in LEWzizi animals to brain regions that are affected by pre-existing LEWzizi-related pathologies.

### Despite major differences in the state of pre-activation, the EAE-induced macrophage/microglia response is similar between Lewis and LEWzizi animals

**Fig. 4:**
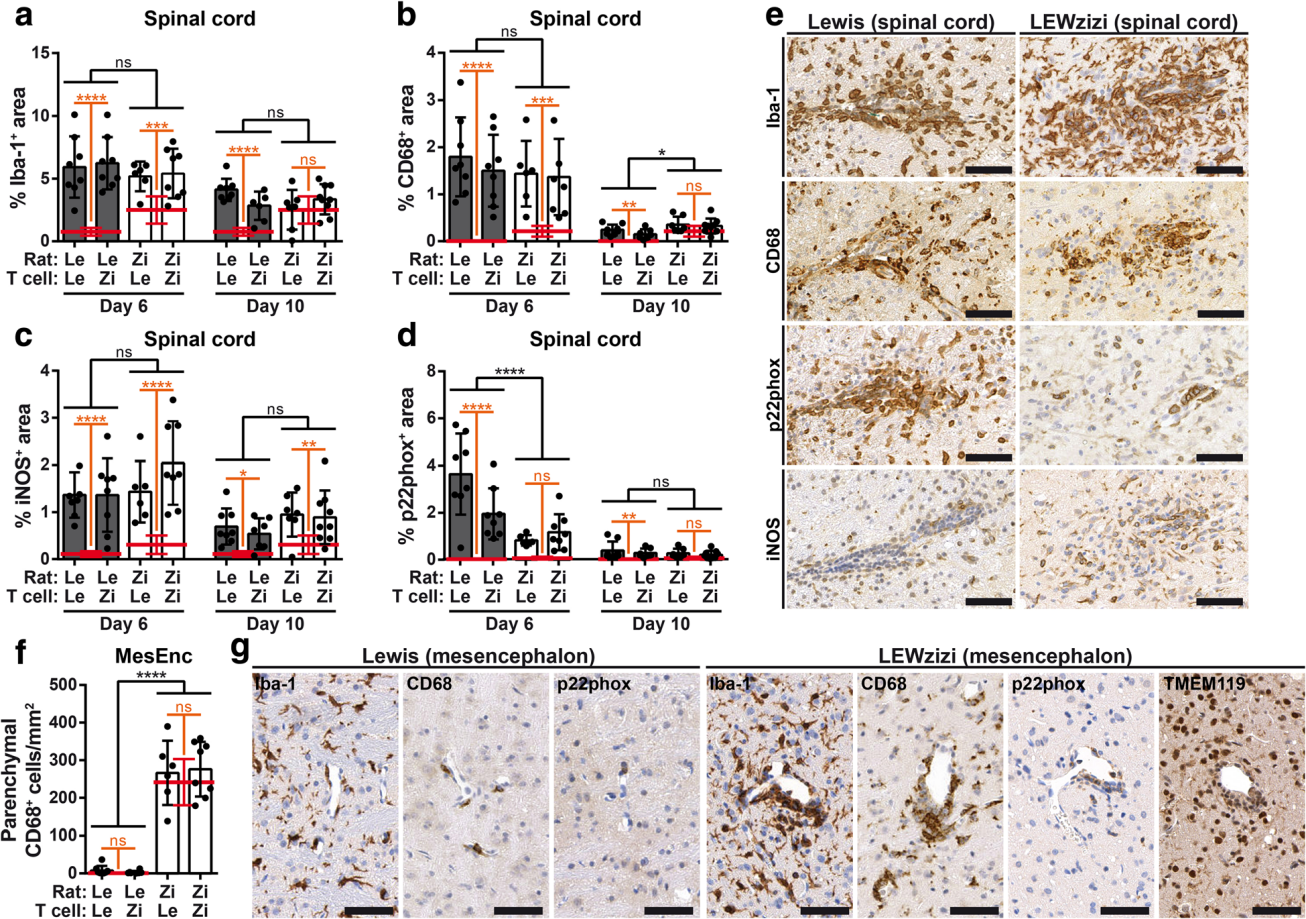
EAE-induced microglia/macrophage response is not amplified in the LEWzizi CNS despite pre-existing microglia activation. **a**-**d** Area fraction analysis of Iba-1 (**a**), CD68 (**b**), iNOS (**c**) and p22phox (**d**) immunohistochemical stainings of lumbar spinal cord cross sections of 4-month-old (4 M) Lewis and LEWzizi rats at the peak (day 6) and during the recovery phase (day 10) of EAE. The percentage of positively labelled area is depicted. **e** Inflammatory lesions with perivascular cuffs and parenchymal infiltrates stained for Iba-1, CD68, p22phox and iNOS. Representative pictures were taken from lumbar spinal cord cross sections of 4 M Lewis and LEWzizi rats, both injected with Lewis T cells, at the peak of EAE. Scale bars, 50 μm. **f** Quantification of parenchymal CD68^+^ cells within the mesencephalon of 4 M Lewis and LEWzizi rats at the peak of EAE. **g** Representative areas in the mesencephalon of 4 M Lewis and LEWzizi rats, each injected with Lewis T cells, at the peak of EAE. Pictures were taken from tissue sections stained for Iba-1, CD68, p22phox and TMEM119 (LEWzizi only). Scale bars, 50 μm. **a**-**d**; **f** Graphs represent mean ± SD. Red lines indicate the mean ± SD of age-matched naïve Lewis or LEWzizi controls. Experimental groups comprise 6–8 rats each. Statistics result from two-way ANOVAs (separate analyses for day 6 and day 10) reporting (i) differences between Lewis EAE rats and LEWzizi EAE rats by black bars and (ii) differences between naïve controls rats and EAE rats by orange bars. Data were pooled according to rat genotype and independent of T cell genotype. *, *p*-value < 0.05; **, *p*-value < 0.01; ***, *p*-value < 0.001; ****, *p*-value < 0.0001; ns, not significant

Although number and activation status of microglia were already significantly elevated in naïve LEWzizi rats, we found an overall similar expression levels of the pan-microglia/macrophage marker Iba-1 and the activation markers CD68 and iNOS in Lewis and LEWzizi recipients at day 6 post EAE induction (Fig. 4a-c, e; Additional file 1: Figure S5e-g; expression levels of naïve controls are indicated by red bars). However, the magnitude of increase in Iba-1 immunoreactivity between naïve controls and EAE day 6 was significantly lower in LEWzizi compared with Lewis rats (interaction effect determined by two-way ANOVA; 4 M, *p* = 0.0114; 8 M, *p* = 0.0008). Also, antibody labelling for p22phox at day 6 was significantly decreased in LEWzizi EAE spinal cords (Fig. 4d; Additional file 1: Figure S5 h). In rodents, p22phox immunoreactivity is usually absent from microglia and solely found on meningeal/perivascular cells and blood monocytes [48]. During EAE in both 4 M and 8 M Lewis rats, p22phox expression increased due to monocyte/macrophage infiltration; this increase was, however, significantly lower in 4 M LEWzizi animals (interaction effect: *p* = 0.0035). In the mesencephalon, a brain area for which we observed a LEWzizi-specific increase in CD3^+^ inflammatory cuffs (Fig. 3c), the numbers of parenchymal CD68^+^ cells did not further increase upon EAE induction in Lewis or LEWzizi rats (Fig. 4f; Additional file 1: Figure S5i). In comparison with the spinal cord, perivascular and parenchymal accumulation of p22phox^+^ cells was not as pronounced in the mesencephalon of both genotypes (Fig. 4g). Statistical testing revealed that only the genetic background of the recipient rats influenced the macrophage/microglia responses, while T cells derived from both Lewis and LEWzizi rats elicited comparable responses (Additional file 1: Table S1).

Expression analysis of microglia/macrophage-associated genes in lumbar spinal cord homogenates confirmed our neuropathological findings that (i) the extent of neuroinflammation in 4 M and 8 M MBP-EAE rats resemble each other and (ii) LEWzizi rats presented with a predominantly dampened neuroinflammatory response (Additional file 1: Figure S6).

### MBP-EAE in LEWzizi rats does not worsen oligodendrocyte and myelin pathology, while axonal damage shows a region-specific modulation

**Fig. 5:**
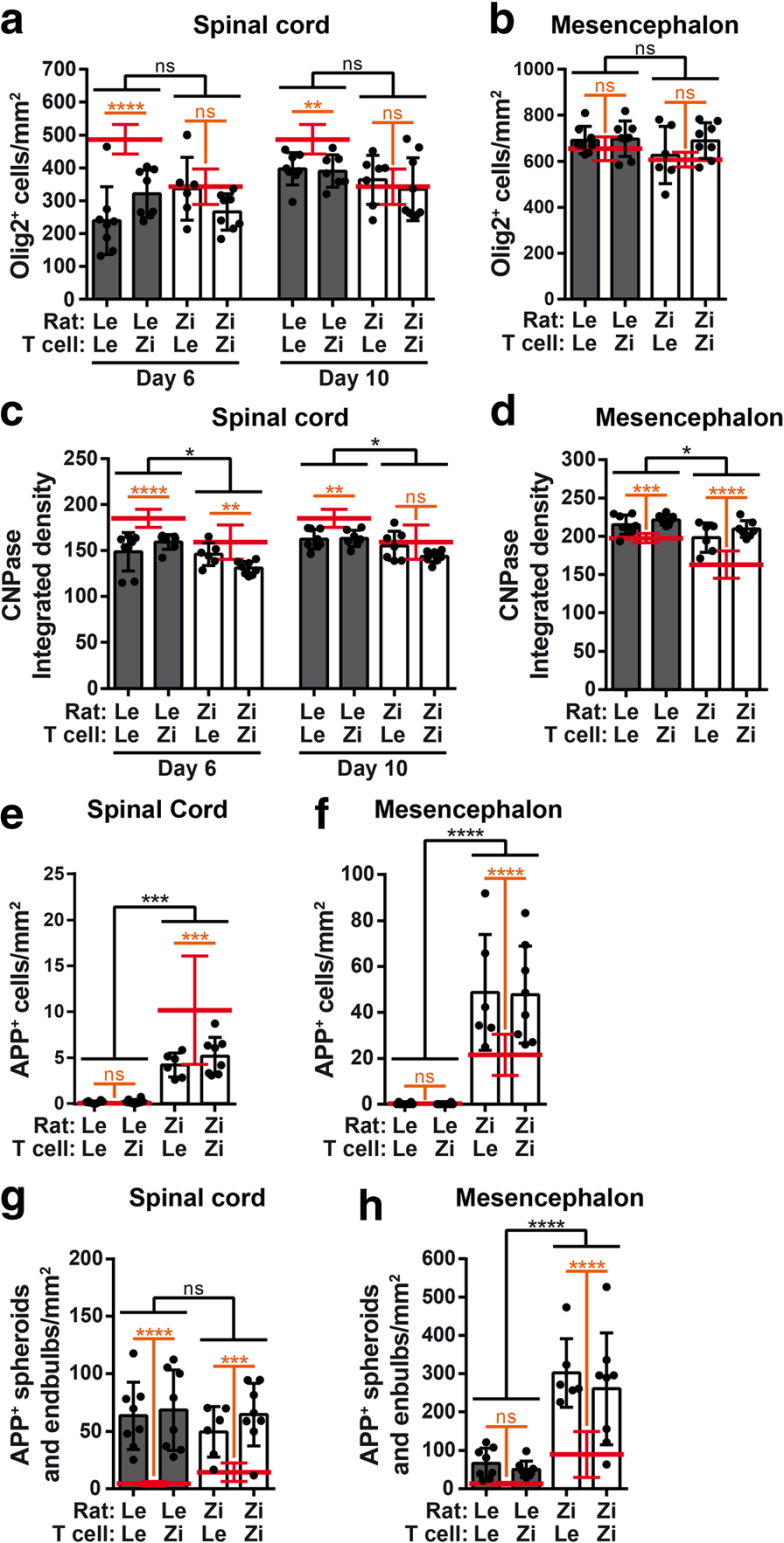
MBP-EAE in LEWzizi mostly influences axonal damage but not pre-existing oligodendrocyte/myelin pathology. **a**, **b** Quantification of immunohistochemical stainings for Olig2 in the grey matter of the lumbar spinal cord (**a**) and mesencephalon (**b**) of 4-month-old (4 M) Lewis and LEWzizi rats at the peak (day 6; **a**, **b**) and during the recovery phase (day 10; **a**) of EAE. **c, d** Level of myelination assessed by densitometric analysis measuring integrated density of CNPase antibody labelling in the grey matter of the lumbar spinal cord (**c**) and mesencephalon (d) of 4 M Lewis and LEWzizi rats at day 6 (**c**, **d**) and day 10 (**c**) of EAE. **e-h** Quantification of cells with strong cytoplasmic accumulation of APP (**e**, **f**) or APP-positive neuronal spheroids and endbulbs (g, **h**) in the grey matter of the lumbar spinal cord (**e**, **g**) and mesencephalon (**f**, **h**) of 4 M Lewis and LEWzizi rats at the peak (day 6) of EAE. Graphs represent mean ± SD. Red lines indicate the mean ± SD of age-matched naïve Lewis or LEWzizi controls. Experimental groups comprise 6–8 rats each. Statistics result from two-way ANOVAs (separate analyses for day 6 and day 10) reporting (i) differences between Lewis EAE rats and LEWzizi EAE rats by black bars and (ii) differences between naïve controls rats and EAE rats by orange bars. Data were pooled according to rat genotype and independent of T cell genotype. *, *p*-value < 0.05; **, *p*-value < 0.01; ***, *p*-value < 0.001; ****, *p*-value < 0.0001; ns, not significant

Similarly as for microglia/macrophages, MBP-EAE did not greatly exacerbate pre-existing oligodendrocyte and myelin pathology in the LEWzizi spinal cord. As expected, induction of EAE led to significantly decreased oligodendrocyte numbers in the spinal cord grey matter of Lewis rats (Fig. 5a; Additional file 1: Figure S5j; expression levels of naïve controls are indicated by red bars). In LEWzizi rats, EAE hardly caused a reduction of Olig2^+^ cells, so that the total density of positively stained cells was similar in both genotypes after EAE induction (Fig. 5a; Additional file 1: Figure S5j). In the mesencephalon, induction of EAE did not lead to a reduction of oligodendrocyte counts in both Lewis and LEWzizi rats (Fig. 5b; Additional file 1: Figure S5n). Generally, passive MBP-EAE does not result in distinct demyelinated lesions; however, myelin density in lumbar spinal cords is usually slightly reduced upon induction of neuroinflammation. This was also observed in our experiments. Starting with already decreased myelin density in naïve LEWzizi rats, the level of myelination stayed constantly lower in the LEWzizi spinal cord compared with Lewis counterparts (interaction effects at day 6: 4 M, *p* = 0.2897; 8 M, *p* = 0.7136) (Fig. 5c; Additional file 1: Figure S5k). However, the decrease was significantly more pronounced in the mesencephalon of 4 M LEWzizi (interaction effect *p* = 0.0132) (Fig. 5d; Additional file 1: Figure S5o).

Interestingly, the number of APP-positive oligodendrocytes, indicative of cells putatively facing severe cellular stress [2], in spinal cord cross sections of LEWzizi rats decreased upon EAE induction (Fig. 5e; Additional file 1: Figure S5 l). The opposite was observed in the mesencephalon (Fig. 5f; Additional file 1: Figure S5p). Axonal injury increased in both the spinal cord and brain of 4 M and 8 M Lewis and LEWzizi rats injected with MBP-T cells (Fig. 5g, h; Additional file 1: Figure S5 m, q). However, although APP-positive neuronal spheroids and endbulbs reached comparable levels at day 6 post disease induction in the lumbar spinal cords of Lewis and LEWzizi rats, there was an increase in axonal tissue injury in LEWzizi brains (Fig. 5g, h; Additional file 1: Figure S5 m, q). Similarly as for the neuroinflammatory response, only the genotype of the recipient rats but not the genetic background of the transferred T cells influenced myelin and axonal pathology (Additional file 1: Table S1).

On the whole, induction of MBP-EAE in a pre-injured CNS environment did not cause a general exacerbation of microglia/macrophage activation and infiltration, oligodendrocyte/myelin pathology as well as axonal damage, although region-specific re-direction of some neuroinflammatory and degenerative processes was noted.

## Discussion

In the present study, we aimed to investigate whether pre-existing microglia activation, iron accumulation and neurodegeneration, which are conditions seen in human brain aging [58] and particularly in the normal-appearing white matter of patients with progressive multiple sclerosis [34], amplify experimentally induced neuroinflammation and tissue injury in the course of passive EAE. Additionally, we intended to test, whether acute monophasic T cell-mediated EAE is transformed into a chronic progressive course, when occurring on such a pre-injured background. To this end, we crossed the outbred zitter rat model to inbred Lewis rats, which are highly susceptible to the induction of EAE, thereby generating so-called LEWzizi rats. Like zitter rats [14, 16, 18, 19, 49], LEWzizi rats present with massive microgliosis accompanied by a general pro-inflammatory environment and with hypomyelination associated with aberrant myelin sheaths, reduced numbers of myelinated fibres, decreased oligodendrocyte densities and the presence of myelin degradation products within phagocytes. Similarly to zitter rats [14, 52, 53, 55, 56], we observed neurodegenerative processes, as shown by the accumulation of APP within neuronal spheroids and endbulbs, in naïve LEWzizi brains and spinal cords.

During normal aging in humans, iron accumulates in oligodendrocytes and axons [12]. In disease conditions, excessive iron accumulation can pose a major problem. When its levels exceed endogenous storage capacities or when it is liberated due to cellular stress and cell death, iron can potentiate oxidative stress via the Fenton reaction [59]. A general pitfall of rodent models of human diseases is their very low iron load; thus, a crucial co-factor for the study of neuroinflammation or neurodegeneration is absent in rodent-based experiments [48]. This can be circumvented by studying LEWzizi rats, which, similarly to zitter rats [49], present with age-dependently increasing, abnormally high iron levels within axonal tracts, oligodendrocytes (brain only) and microglia (both brain and spinal cord). Moreover, they show signs of oxidative stress, which had previously been comprehensively described for zitter rats as well [11, 14, 36, 54, 55]. Here, we used a histopathological approach and observed high numbers of LEWzizi microglia expressing iNOS. However, we could not detect any parenchymal p22phox expression in the whole CNS, in line with previous reports involving normal and inflamed rodent brains [48].

Taken together, LEWzizi rats represent a suitable model to study the consequences of experimentally induced neuroinflammation on a background of microglia activation, oxidative injury, neurodegeneration and iron accumulation. Induction of EAE by passive transfer of MBP-specific CD4^+^ T cells in both Lewis and LEWzizi rats led to typical monophasic EAE. Disease scores were slightly, but significantly higher in LEWzizi compared with Lewis rats, which, however, may have resulted from an additive effect of LEWzizi- and EAE-related clinical signs [42]. Clinical appearance of EAE manifests through lesions in the spinal cord and medulla oblongata, while lesions in the forebrain do not impact clinical scores. In LEWzizi rats, passive EAE started slightly, but significantly earlier than in Lewis controls. We suspect that the early start of disease resulted from the increased pro-inflammatory milieu in LEWzizi spinal cords. Initiation of EAE after induction of focal injuries reportedly reduced the threshold for lesion formation in previous studies [29].

Our neuropathological analyses clearly showed that T cell infiltration patterns significantly differed between wild-type Lewis and LEWzizi rats. Similarly to Lewis rats, T cell infiltrates were observed in lumbar spinal cords, the predilection site for classical MBP-EAE [1], of LEWzizi rats, although they were less abundant. On the contrary, T cells were detected at sites in the CNS, which are usually not targeted in Lewis controls, such as the deep brain stem nuclei including the mesencephalon. We believe that this difference in the topography of T cell infiltration is due to the underlying pro-inflammatory and neurodegenerative LEWzizi pathology, since T cell re-distribution specifically targeted pre-damaged areas. Similar re-distribution phenomena of EAE lesions have already been described earlier: Focal injuries to rat brains induced by burns, cryogenic insults, chemicals or anoxic injuries prior to active sensitization with a CNS antigen or passive transfer of CNS-reactive T cells led to the formation of EAE lesions in the pre-damaged forebrain areas, which were barely targeted in control rats [7, 21, 23, 27, 28, 30, 31, 40]. Additionally, it has been shown in mice that induction of forebrain neurodegeneration by a cuprizone-supplemented diet followed by active or passive MOG-EAE resulted in immune cell recruitment to areas of the brain parenchyma that are usually not targeted in naïve animals [4, 44, 47]. Interestingly, in contrast to the brain, LEWzizi-specific pre-damage in the spinal cord did not lead to enhanced T cell recruitment into this particular CNS area. Instead, significantly reduced numbers of T cell cuffs and total CD3^+^ immunoreactivity were observed upon disease induction by CD4^+^ T cells.

The role of microglia in EAE has been studied previously in transgenic mouse models. Stalling of microglia proliferation massively dampened clinical severity and neuropathology [15]. Restricting microglia activation abolished clinical disease and strongly diminished neuroinflammation and tissue destruction [10]. Microglia ablation via Csf1R inhibition at the onset of disease massively dampened clinical severity and greatly reduced EAE pathology [38]. Reduction of microglia reactivity by dipyridamole attenuated clinical as well as histological EAE scores [50]. Similarly, application of minocycline or microglia/macrophage inhibitory factor TKP attenuated EAE [6, 41]. Contrary, other previous studies have suggested that microglia pre-activation does not interfere with autoimmune disease induced by CD4^+^ T cells. Ablation of microglia in a CX3CR1-transgenic mouse model leads to a rapid repopulation of microglia from remaining progenitor cells resulting in type-1-interferon-pathway-related microglia activation associated with neurodegeneration [43]. In that study, induction of EAE at the peak of microglia activation did not change T cell-mediated inflammation, neurodegeneration or demyelination. Similarly, induction of EAE in animals with active cuprizone lesions resulted in the precipitation of inflammatory lesions at the sites of cuprizone-induced demyelination, but neither cuprizone-related demyelination and neurodegeneration nor EAE-related neurodegeneration were altered [44]. Here in our study, we found that microglia pre-activation in LEWzizi rats did not exacerbate tissue injury in CD4^+^ T cell-induced EAE. Although naïve LEWzizi animals initially had high numbers of activated microglia, the levels of CD68^+^ phagocytes (microglia and peripheral macrophages) were not increased at the peak of EAE compared with Lewis rats. Likewise, the number of Iba^+^ phagocytes, which was strongly elevated in naïve LEWzizi rats, was either equal or lower at the peak of EAE compared with Lewis animals. The phagocyte activation markers iNOS and p22phox as well as APP-positive neuronal spheroids and endbulbs (representing neuronal injury and damage, respectively) followed a rather similar pattern.

## Conclusion

Taken together, we could collect in our study interesting and unexpected data that were in contrast to our initial key hypotheses. Importantly, we observed that tissue damage induced by CD4^+^ T cell-mediated inflammation was not aggravated, when induced on the LEWzizi-specific background of microglia pre-activation, cellular iron accumulation, diffuse myelin damage and neurodegeneration. Also, EAE did not convert into a chronic progressive inflammatory disease. In view of previously reported data, these findings were rather unexpected. For example, tissue injury in progressive MS is described to depend on chronic inflammation comprising T and B cells and occurs on the background of microglia activation, iron accumulation and neurodegeneration [34], conditions similar to those in our LEWzizi model. A possible explanation for this discrepancy may reside in differences in the inflammatory process between MS and EAE. Most EAE models, including the one used in our experiments, are driven by MHC class II-restricted CD4^+^ T cells [24]. However, in MS and particularly in patients with active progressive disease, the inflammatory reaction mainly consists of tissue-resident CD8^+^ memory T cells and B cells [33, 57]. Thus, future studies will be necessary to determine the interaction of these inflammatory cell populations with microglia activation and tissue damage.

## Additional file

Additional file 1: Supplementary Methods. **Table S1.** Possible influences of the two independent variables rat genotype and T cell genotype (and possible interaction between them) on the investigated parameters tested via two-way ANOVAs. **Table S2.** Pathway analysis of differentially expressed genes. **Table S3.** Description of the employed TaqMan assays. **Figure S1.** Microgliosis in the LEWzizi CNS. **Figure S2.** Gene expression analysis of microglia-associated genes. **Figure S3.** Astrocytosis and iron accumulation in the LEWzizi CNS. **Figure S4.** Oligodendrocyte, myelin and axonal pathologies in the LEWzizi CNS. **Figure S5.** Quantification of neuroinflammation, myelin pathology and neuronal damage in MBP-EAE rats. **Figure S6.** Gene expression profiling in spinal cord tissue from EAE rats. (PDF 7049 kb)

## Abbreviations

CNS: Central nervous system
EAE: Experimental autoimmune encephalomyelitis
MS: Multiple sclerosis
TBB: Turnbull Blue
