# Supplementary material for: Microglia pre-activation and neurodegeneration precipitate neuroinflammation without exacerbating tissue injury in experimental autoimmune encephalomyelitis
Source: Acta Neuropathol Commun. 2019 Jan 31;7:14. doi: 10.1186/s40478-019-0667-9 (PMC6357376; doi:10.1186/s40478-019-0667-9)
Supplement: Supplementary file 1 — Supplementary Methods. Table S1. Possible influences of the two independent variables rat genotype and T cell genotype (and possible interaction between them) on the investigated parameters tested via two-way ANOVAs. Table S2. Pathway analysis of differentially expressed genes. Table S3. Description of the employed TaqMan assays. Figure S1. Microgliosis in the LEWzizi CNS. Figure S2. Gene expression analysis of microglia-associated genes. Figure S3. Astrocytosis and iron accumulation in the LEWzizi CNS. Figure S4. Oligodendrocyte, myelin and axonal pathologies in the LEWzizi CNS. Figure S5. Quantification of neuroinflammation, myelin pathology and neuronal damage in MBP-EAE rats. Figure S6. Gene expression profiling in spinal cord tissue from EAE rats. (PDF 7049 kb) [file 40478_2019_667_MOESM1_ESM.pdf]

Supplementary Data

Microglia pre-activation and neurodegeneration precipitate neuroinflammation without exacerbating tissue injury in experimental autoimmune encephalomyelitis

Isabella Wimmer[1], Cornelia Scharler[1], Tobias Zrzavy[1], Taro Kadowaki[1, 2], Verena Mödlagl[1], Kim Rojc[1], Anna R. Tröscher[1], Maja Kitic[1], Shuichi Ueda[3], Monika Bradl[1], Hans Lassmann[1]

[1] Department of Neuroimmunology, Center for Brain Research, Medical University of Vienna, Austria

[2] Department of Neurology, Dokkyo Medical University, Tochigi, Japan

[3] Department of Histology and Neurobiology, Dokkyo Medical University, Tochigi, Japan

Supplementary Methods………………………………………………………… 2

Supplementary Tables………………………………………………………… 3

Supplementary Figures………………………………………………………… 7

Supplementary References………………………………………………………… 13

Supplementary Methods

Histological stainings

For antibody double labellings with APP and cell type-specific markers, tissue sections were first incubated with primary anti-APP antibody following the routine staining protocol. On the subsequent day, alkaline phosphatase-conjugated donkey-α-mouse antibody (1:100 in 10% FCS/ Dako buffer; 715-055-151, Jackson ImmunoResearch) was applied and the APP staining was developed with Fast Blue (Bauer and Lassmann, 2016). Tissue sections were again incubated in either citrate or Tris/EDTA buffer (Table 1) and steaming was performed for 30 minutes. The second primary antibodies (Iba-1, Gfap, Olig2 or NeuN) were applied overnight at 4 °C. Antibody labelling for cell type-specific markers was visualized by incubation with biotinylated secondary antibodies and development of the staining with 3-amino-9-ethylcarbazol (AEC) (Bauer and Lassmann, 2016). Tissue sections were mounted in geltol. Double stainings for CD68 with P2RY12 or MHC class II were done likewise.

For fluorescent double labelling (iNOS + Iba-1 and iNOS + GFAP), tissue blocks were routinely cut (3 µm), deparaffinized in xylene and rinsed in 96% ethanol. Subsequently, endogenous peroxidase activity was blocked in MeOH/ 0.2% H_2O_2 for at least 20 minutes and tissue sections were rehydrated. Antigen retrieval was performed for 1 hour in a commercial food steamer using 10 mM citrate buffer (pH 6.0). Thereafter, tissue sections were incubated in Dako REAL Antibody Diluent (S3006, Dako) for at least 20 minutes to block non-specific binding. Primary antibody against iNOS (1:7,500 in Antibody Diluent; modified factor compared to Table 1 due to signal enhancement) was applied and incubated overnight at 4 °C. Biotinylated donkey-α-rabbit (711-065-152, Jackson ImmunoResearch) was diluted 1:2,000 in 10% FCS/ Dako buffer and applied for 1 hour at room temperature (RT). Thereafter, tissue sections were incubated with peroxidase-conjugated streptavidin (1:500 in 10% FCS/ Dako buffer; 016-030-084, Jackson ImmunoResearch) for 1 hour at RT followed by a catalyzed signal amplification (CSA) step, for which tissue sections were incubated with biotinylated tyramine [1,7] for 20 minutes. Then, tissue sections were again steamed for 30 minutes in 1 mM EDTA/10 mM Tris buffer (pH 8.6) followed by incubation with Cy2-conjugated streptavidin (1:100 in 10% FCS/ Dako buffer; 016-220-084, Jackson ImmunoResearch) to visualize iNOS antibody labelling. Subsequently, tissue sections were incubated with primary antibodies against either Iba-1 or GFAP (both 1:3,000 in 10% FCS/ Dako buffer; Table 1) overnight at 4 °C. On the third day, Cy3-conjugated donkey-α-rabbit secondary antibodies (1:200 in 10% FCS/ Dako buffer; 711-165-152, Jackson ImmunoResearch) were applied and tissue sections were mounted in ProLong mounting medium.

For double labelling of Iba-1 with luxol fast blue myelin stain (LFB), conventional DAB staining for Iba-1 was performed as described in the Materials and Methods section. Thereafter, the LFB staining procedure was followed according to routine protocols.

TaqMan qPCR

Using the High Capacity cDNA Reverse Transcription Kit (Thermo Fisher Scientific), 500 ng total RNA were transcribed into cDNA according to the manufacturer's instructions. Thereof, 400 ng RNA equivalents per sample were mixed with TaqMan™ Gene Expression Master Mix (Thermo Fisher Scientific) and loaded into each reservoir of Custom 384-Well Microfluidic Card TaqMan Gene Expression Arrays (Thermo Fisher Scientific). According to the manufacturer's protocols, microfluidic cards were centrifuged, sealed and run at a 7900HT Fast Real-Time PCR System employing SDS Software v2.4 (Applied Biosystems). Data were analysed using ExpressionSuite Software v1.0.3 (Thermo Fisher Scientific). The comparative CT (ΔΔCT) method was used to calculate relative quantities (RQ). Data normalization, as conducted by the analysis software, was based on five reference genes: 18s ribosomal RNA (*18S*), glyceraldehyde-3-phosphate dehydrogenase (*Gapdh*), hypoxanthine phosphoribosyltransferase 1 (*Hprt1*), phosphoglycerate kinase 1 (*Pgk1*), peptidylprolyl isomerase A (*Ppia*). A detailed description of the employed TaqMan assays is provided in Supplementary Table 3.

Supplementary Tables

Supplementary Table 1: Possible influences of the two independent variables rat genotype and T cell genotype (and possible interaction between them) on the investigated parameters tested via two-way ANOVAs.

Corresponding Figure	Investigated Parameter	p-value Rat genotype	p-value T cell genotype	p-value Interaction
**Fig. 2c**	**Score at peak of disease** (4M)	0.0089	0.2335	0.2242
	**Score at peak of disease** (8M)	0.0013	0.8662	0.0142
**Fig. 2d**	**Day of EAE onset** (4M)	0.0002	0.6543	0.3194
	**Day of EAE onset** (8M)	< 0.0001	0.6741	0.9742
**Fig. 3a**	**CD3+ cuffs** (SpC, 4M, day6)	0.0002	0.7657	0.9110
**Fig. 3b**	**% CD3+ area** (SpC, 4M, day6)	<0.0001	0.1896	0.2024
**Fig. 3c**	**CD3+ cuffs** (MesEnc, 4M, day6)	n.a.	n.a.	n.a.
**Fig. 3d**	**CD3+ cells** (MesEnc, 4M, day6)	<0.0001	0.4719	0.6743
**Sup. Fig. 7a**	**CD3+ cuffs** (SpC, 8M, day6)	0.0003	0.0436	0.6794
**Sup. Fig. 7b**	**CD3+ cuffs** (MesEnc, 8M, day6)	n.a.	n.a.	n.a.
**Sup. Fig. 7c**	**% CD3+ area** (SpC, 8M, day6)	0.0054	0.1929	0.6422
**Sup. Fig. 7d**	**CD3+ cells** (MesEnc, 8M, day6)	0.0019	0.5178	0.6666
**Fig. 4a**	**% Iba-1+ area** (SpC, 4M, day6)	0.3003	0.7240	0.9642
	**% Iba-1+ area** (SpC, 4M, day10)	0.2300	0.6224	0.0239
**Fig. 4b**	**% CD68+ area** (SpC, 4M, day6)	0.4034	0.5320	0.7005
	**% CD68+ area** (SpC, 4M, day10)	0.0087	0.2424	0.5486
**Fig. 4c**	**% iNOS+ area** (SpC, 4M, day6)	0.1700	0.2635	0.2576
	**% iNOS+ area** (SpC, 4M, day10)	0.0754	0.5259	0.7713
**Fig. 4d**	**% p22phox+ area** (SpC, 4M, day6)	0.0002	0.1202	0.0218
	**% p22phox+ area** (SpC, 4M, day10)	0.3088	0.3616	0.8139
**Fig. 4f**	**CD68+ cells** (MesEnc, 4M, day6)	<0.0001	0.9074	0.7053
**Sup. Fig. 7e**	**% Iba-1+ area** (SpC, 8M, day6)	0.0002	0.8376	0.3667
	**% Iba-1+ area** (SpC, 8M, day10)	0.7024	0.7040	0.3517
**Sup. Fig. 7f**	**% CD68+ area** (SpC, 8M, day6)	0.4559	0.4841	0.6255
	**% CD68+ area** (SpC, 8M, day10)	0.5166	0.4337	0.7564
**Sup. Fig. 7g**	**% iNOS+ area** (SpC, 8M, day6)	0.1647	0.8196	0.9093
	**% iNOS+ area** (SpC, 8M, day10)	0.2115	0.2688	0.7322
**Sup. Fig. 7h**	**% p22phox+ area** (SpC, 8M, day6)	0.0123	0.5590	0.9340
	**% p22phox+ area** (SpC, 8M, day10)	0.0198	0.0318	0.7233
**Sup. Fig. 7i**	**CD68+ cells** (MesEnc, 8M, day6)	<0.0001	0.8375	0.9434
**Fig. 5a**	**Olig2+ cells** (SpC GM, 4M, day6)	0.4999	0.8473	0.0186
	**Olig2+ cells** (SpC GM, 4M, day10)	0.0969	0.4971	0.6753
**Fig. 5b**	**Olig2+ cells** (MesEnc, 4M, day6)	0.2500	0.2772	0.3667
**Fig. 5c**	**CNPase IntDen** (SpC GM, 4M, day6)	0.0039	0.6517	0.0144
	**CNPase IntDen** (SpC GM, 4M, day10)	0.0017	0.1790	0.1205
**Fig. 5d**	**CNPase IntDen** (MesEnc, 4M, day6)	0.0051	0.0769	0.5908
**Fig. 5e**	**APP+ cells** (SpC, 4M, day6)	<0.0001	0.2568	0.3162
**Fig. 5f**	**APP+ cells** (MesEnc, 4M, day6)	<0.0001	0.9239	0.9418
**Fig. 5g**	**APP+ spheroids** (SpC, 4M, day6)	0.4137	0.3608	0.6440
**Fig. 5h**	**APP+ spheroids** (MesEnc, 4M, day6)	<0.0001	0.3870	0.7043

**Sup. Fig. 9a**	**CNPase+ cells** (SpC GM, 4M, day6)	0.6028	0.1200	0.4089
	**CNPase+ cells** (SpC GM, 4M, day10)	0.0510	0.7092	0.5400
**Sup. Fig. 9b**	**CNPase+ cells** (SpC GM, 8M, day6)	0.8028	0.0304	0.1389
	**CNPase+ cells** (SpC GM, 8M, day10)	0.0667	0.1002	0.6846
**Sup. Fig. 9c**	**CNPase+ cells** (MesEnc, 4M, day6)	0.0121	0.7288	0.6682
**Sup. Fig. 9d**	**CNPase+ cells** (MesEnc, 8M, day6)	0.8968	0.1263	0.2980
**Sup. Fig. 9e**	**Olig2+ cells** (SpC GM, 8M, day6)	0.0454	0.2189	0.5370
	**Olig2+ cells** (SpC GM, 8M, day10)	<0.0001	0.7177	0.6439
**Sup. Fig. 9f**	**CNPase IntDen** (SpC, 8M, day6)	0.0453	0.3403	0.8909
	**CNPase IntDen** (SpC, 8M, day10)	0.3122	0.1071	0.3685
**Sup. Fig. 9g**	**APP+ cells** (SpC, 8M, day6)	<0.0001	0.7980	0.8084
**Sup. Fig. 9h**	**APP+ spheroids** (SpC, 8M, day6)	0.0927	0.8274	0.5583
**Sup. Fig. 9i**	**Olig2+ cells** (MesEnc, 8M, day6)	0.4384	0.9343	0.7538
**Sup. Fig. 9j**	**CNPase IntDen** (MesEnc, 8M, day6)	<0.0001	0.2717	0.3656
**Sup. Fig. 9k**	**APP+ cells** (MesEnc, 8M, day6)	<0.0001	0.3863	0.3932
**Sup. Fig. 9l**	**APP+ spheroids** (MesEnc, 8M, day6)	<0.0001	0.7306	0.8503

n.a., statistic not available (at least one experimental group contained only equal values, thus analysis of variances is not possible)

Supplementary Table 2: Pathway analysis of differentially expressed genes

Reactome pathway	FDR p-value	Enrichment (all)
Cholesterol biosynthesis	0.00000017	16.65
Metabolism of lipids and lipoproteins	0.00000047	2.75
Metabolism	0.00000152	1.90
Immune System	0.00000163	2.15
Innate Immune System	0.00001120	2.55
Hemostasis	0.00002450	2.70
Platelet activation, signaling and aggregation	0.00017700	3.33
Activation of gene expression by SREBF (SREBP)	0.00046400	11.10
Adaptive Immune System	0.00080700	2.33
Olfactory Signaling Pathway	0.00095800	0.06
Cell surface interactions at the vascular wall	0.00099300	4.53
Regulation of cholesterol biosynthesis by SREBP (SREBF)	0.00104000	9.19
Generation of second messenger molecules	0.00261000	9.71
Complement cascade	0.00452000	7.01
SLC-mediated transmembrane transport	0.00471000	2.81
Sphingolipid metabolism	0.00479000	4.70
Costimulation by the CD28 family	0.00685000	5.55
GPVI-mediated activation cascade	0.02930000	5.03

GO biological process	FDR p-value	Enrichment (≥ 15 only)
Antigen processing and presentation of exogenous peptide antigen	0.00000004	17.37
Antigen processing and presentation of peptide antigen via MHC class II	0.00000006	20.35
Antigen processing and presentation of peptide or polysaccharide antigen via MHC class II	0.00000012	18.32
Antigen processing and presentation of exogenous peptide antigen via MHC class II	0.00000017	22.20
Regulation of hypersensitivity	0.00296000	15.14
D-aspartate transport	0.00339000	26.64
D-aspartate import across plasma membrane	0.00340000	26.64
Angiogenesis involved in coronary vascular morphogenesis	0.00516000	22.20
D-amino acid transport	0.00737000	19.03
Positive regulation of hypersensitivity	0.01010000	16.65
Positive regulation of type II hypersensitivity	0.02190000	24.98
Regulation of type II hypersensitivity	0.02190000	24.98
Positive regulation of type IIa hypersensitivity	0.02200000	24.98
Regulation of type IIa hypersensitivity	0.02200000	24.98
Regulation of T cell activation via T cell receptor contact with antigen bound to MHC molecule on antigen presenting cell	0.02210000	24.98
Positive regulation of type III hypersensitivity	0.02210000	24.98
Regulation of type III hypersensitivity	0.02210000	24.98
Peptide antigen assembly with MHC class II protein complex	0.02220000	24.98
MHC class II protein complex assembly	0.02220000	24.98
Toll-like receptor 7 signaling pathway	0.02230000	24.98
Fc receptor mediated stimulatory signaling pathway	0.03140000	19.98
Regulation of type I hypersensitivity	0.03140000	19.98
Peptide antigen assembly with MHC protein complex	0.03150000	19.98
Regulation of phospholipid catabolic process	0.04180000	16.65
Antigen processing and presentation of exogenous peptide antigen via MHC class I	0.04190000	16.65
Positive regulation of platelet activation	0.04190000	16.65
Positive regulation of interferon-beta biosynthetic process	0.04200000	16.65
Membrane protein intracellular domain proteolysis	0.04200000	16.65
MHC protein complex assembly	0.04210000	16.65

698 differentially expressed genes (± 1.2 fold-change; FDR-corrected p-value ≤ 0.05) were subjected to GO-term-based enrichment analysis (http://geneontology.org/page/go-enrichment-analysis) using PANTHER overrepresentation test (release 20171205) and either Reactome version 58 (release 2016-12-07) or GO Ontology database (release 2018-05-21) for pathway annotation. Statistical testing based on Fisher's exact test with FDR multiple test correction. For the analysis of GO biological processes (violet table), only pathways with an enrichment score of ≥ 15 are shown; in total, 713 pathways with FDR-corrected p-value ≤ 0.05 were detected.

Supplementary Table 3: Description of the employed TaqMan assays

Gene Symbol	Probe Name	Amplicon Length	Exon spanning probe	Gene Name	RefSeq Number
**18S**	Hs99999901_s1	187		18S ribosomal RNA	
**Gapdh**	Rn01775763_g1	175		Glyceraldehyde-3-phosphate dehydrogenase	NM_017008
**Hprt1**	Rn01527840_m1	64	x	Hypoxanthine phosphoribosyltransferase 1	NM_012583
**Pgk1**	Rn01474008_gH	142	x	Phosphoglycerate kinase 1	NM_053291
**Ppia**	Rn00690933_m1	149	x	Peptidylprolyl isomerase A	NM_017101
**Aif1**	Rn00574125_g1	126	x	Allograft inflammatory factor 1	NM_017196
**Arg1**	Rn00691090_m1	76	x	Arginase, liver	NM_017134
**Axl**	Rn00627285_m1	63	x	Axl receptor tyrosine kinase, transcript variant 2	NM_001013147
**Cd40**	Rn01423590_m1	138	x	CD40 molecule, TNF receptor superfamily member 5	NM_134360
**Cd68**	Rn01495634_g1	62	x	Cd68 molecule	NM_001031638
**Cd74**	Rn00565062_m1	68	x	Cd74 molecule, major histocompatibility complex, class II invariant chain	NM_013069
**Cd86**	Rn00571654_m1	96	x	CD86 molecule	NM_020081
**Ciita**	Rn01424725_m1	64	x	Class II, major histocompatibility complex, transactivator	NM_053529
**Cyba**	Rn00577357_m1	72	x	Cytochrome b-245, alpha polypeptide	NM_024160
**Cybb**	Rn00576710_m1	77	x	Cytochrome b-245, beta polypeptide	NM_023965
**Il1b**	Rn00580432_m1	74	x	Interleukin 1 beta	NM_031512
**IL6**	Rn01410330_m1	121	x	Interleukin 6	NM_012589
**Klf4**	Rn00821506_g1	77	x	Kruppel-like factor 4 (gut)	NM_053713
**MafB**	Rn00709456_s1	70		V-maf musculoaponeurotic fibrosarcoma oncogene homolog B (avian)	NM_019316
**Mertk**	Rn00576094_m1	74	x	C-mer proto-oncogene tyrosine kinase	NM_022943
**Mrc1**	Rn01487342_m1	62	x	Mannose receptor, C type 1	NM_001106123
**Msr1**	Rn01488115_m1	62	x	Macrophage scavenger receptor 1	NM_001191939
**Ncf1**	Rn00586945_m1	105	x	Neutrophil cytosolic factor 1	NM_053734
**Ncf2**	Rn01759079_m1	71	x	Neutrophil cytosolic factor 2	NM_001100984
**Ncf4**	Rn01505557_m1	59	x	Neutrophil cytosolic factor 4	NM_001127304
**Nos2**	Rn00561646_m1	77	x	Nitric oxide synthase 2, inducible	NM_012611
**Nox1**	Rn00586652_m1	82	x	NADPH oxidase 1	NM_053683
**RT1-Db1**	Rn01429350_m1	119	x	RT1 class II, locus Db1	NM_001008884
**Tgfb1**	Rn00572010_m1	65	x	Transforming growth factor, beta 1	NM_021578
**Tnf**	Rn01525859_g1	92	x	Tumor necrosis factor (TNF superfamily, member 2)	NM_012675

Supplementary Figures

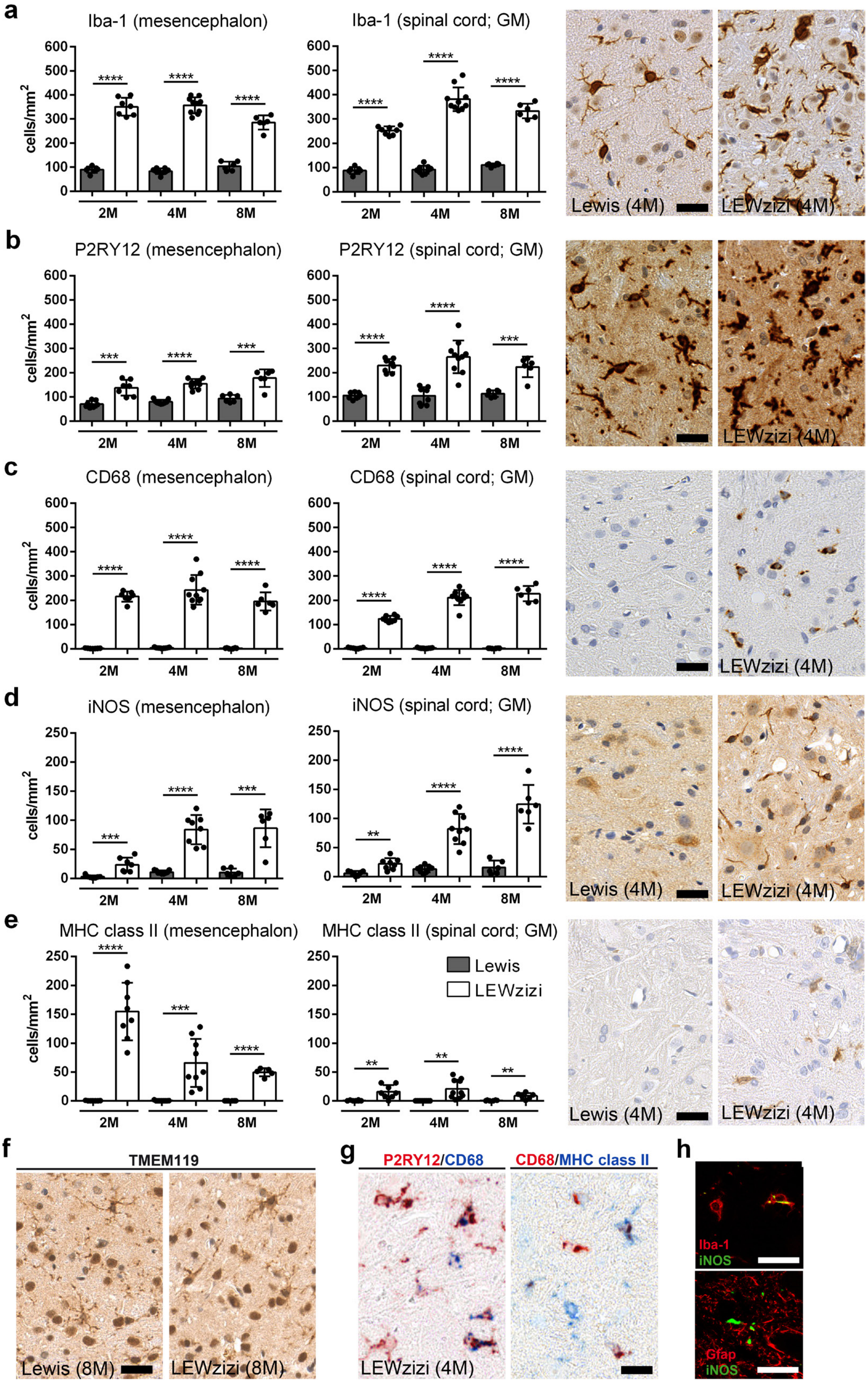

Supplementary Figure 1: Microgliosis in the LEWzizi CNS

(a-e) Quantification and representative pictures of immunohistochemical (IHC) stainings for the pan microglia marker Iba-1 (a), the homeostatic microglia marker P2RY12 (b) and the activation markers CD68 (ED1 clone)(c), iNOS (d) and MHC class II molecule RT1B (e). Only parenchymal cells in the mesencephalon and lumbar spinal cord grey matter (GM) of 2-month-old (2M), 4-month-old (4M) and 8-month-old (8M) Lewis and LEWzizi rats were counted. Representative pictures were taken from the spinal cord GM of 4M Lewis and LEWzizi rats. Bar graphs represent mean ± SD. Data points represent individual rats. Reported statistics result from unpaired, two-tailed Student's t-tests. **, p-value < 0.01; ***, p-value < 0.001; ****, p-value < 0.0001 **(f)** IHC stainings for the microglia-specific marker TMEM119 in the mesencephalon of 8M Lewis and LEWzizi rats **(g)** IHC double stainings for CD68 & P2RY12 and CD68 & MHC class II in the spinal cord GM of 4M LEWzizi rats **(h)** Fluorescent double labelling of iNOS with Iba-1 (microglia) or GFAP (astrocytes) in the spinal cord grey matter of an 8M LEWzizi rat. Scale bars **(a-g)**, 25 µm; scale bar **(h)**, 20 µm

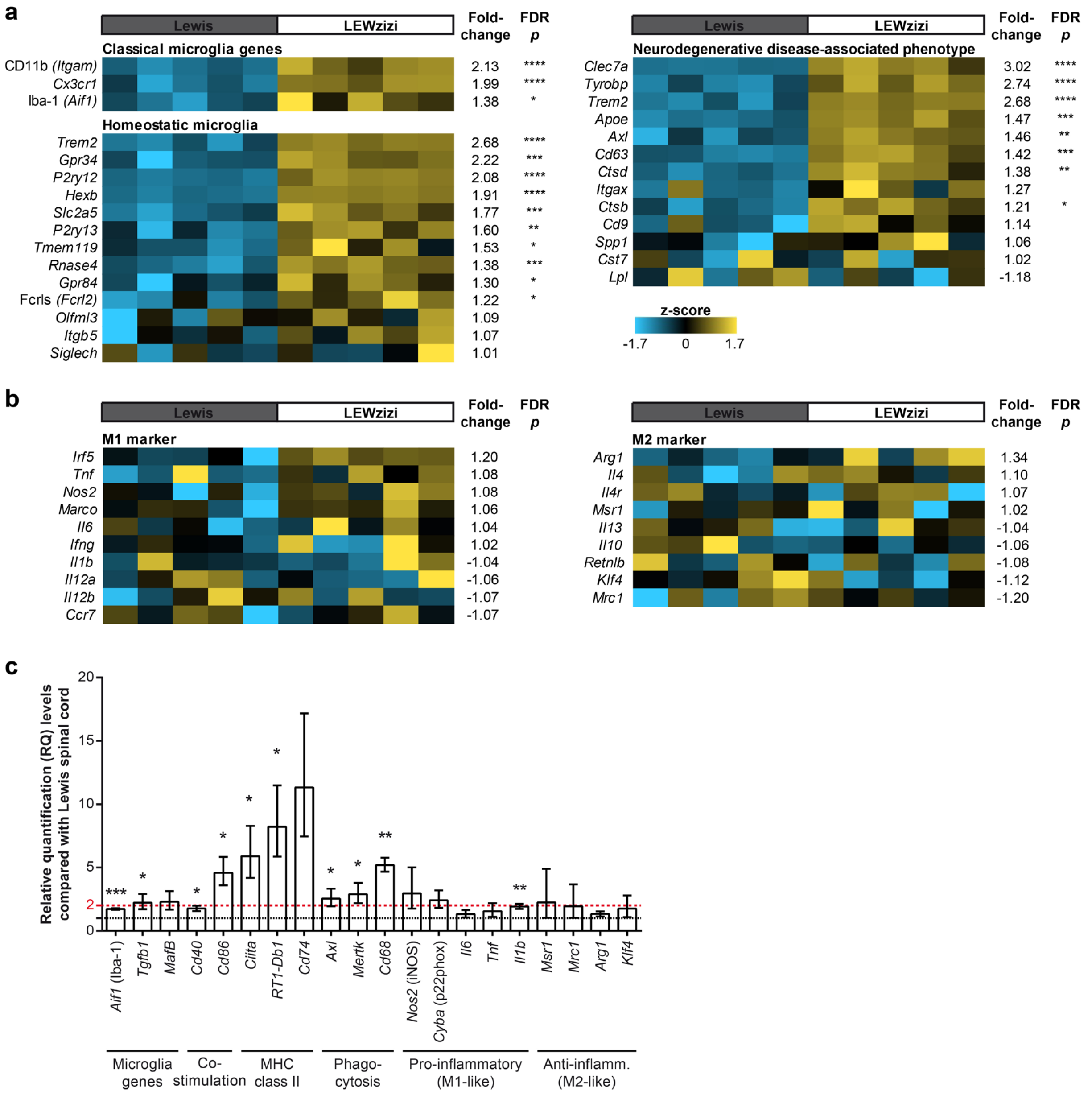

Supplementary Figure 2: Gene expression analysis of microglia-associated genes

(a, b) Z-score analysis of whole-genome microarray data derived from lumbar spinal cord samples of 4-month-old Lewis (n = 5) and LEWzizi (n = 5) rats. Gene signatures for homeostatic and neurodegenerative disease-associated expression patterns derive from previously published data [2,5,3,4,9,6,8]. Reported statistics derive from one-way ANOVAs and Benjamini-Hochberg Step-Up FDR-adjusted p-values. *, p-value < 0.05; **, p-value < 0.01; ***, p-value < 0.001; ****, p-value < 0.0001

(c) Confirmation of gene expression by independent qPCR. TaqMan qPCRs were performed with samples derived from lumbar spinal cord homogenates of 4-month-old Lewis and LEWzizi rats (n = 3 per experimental group). Genes of interest were normalized to five reference genes: 18s ribosomal RNA (*18S*), glyceraldehyde-3-phosphate dehydrogenase (*Gapdh*), hypoxanthine phosphoribosyltransferase 1 (*Hprt1*), phosphoglycerate kinase 1 (*Pgk1*), peptidylprolyl isomerase A (*Ppia*). A detailed description of the employed TaqMan assays is provided in Supplementary Table 3. Data were analysed using ExpressionSuite Software v1.0.3 (Thermo Fisher Scientific) and the calculated fold-changes (RQ, relative quantities) report up- or downregulated gene expression in LEWzizi compared with Lewis samples. Error bars indicate RQMin and RQMax. The red dashed line indicates a 2-fold upregulation of gene expression. The black dotted line indicates a fold-change level of ± 1. Statistics derive from independent Student's t-tests. *, p < 0.05; **, p < 0.01; ***, p < 0.001

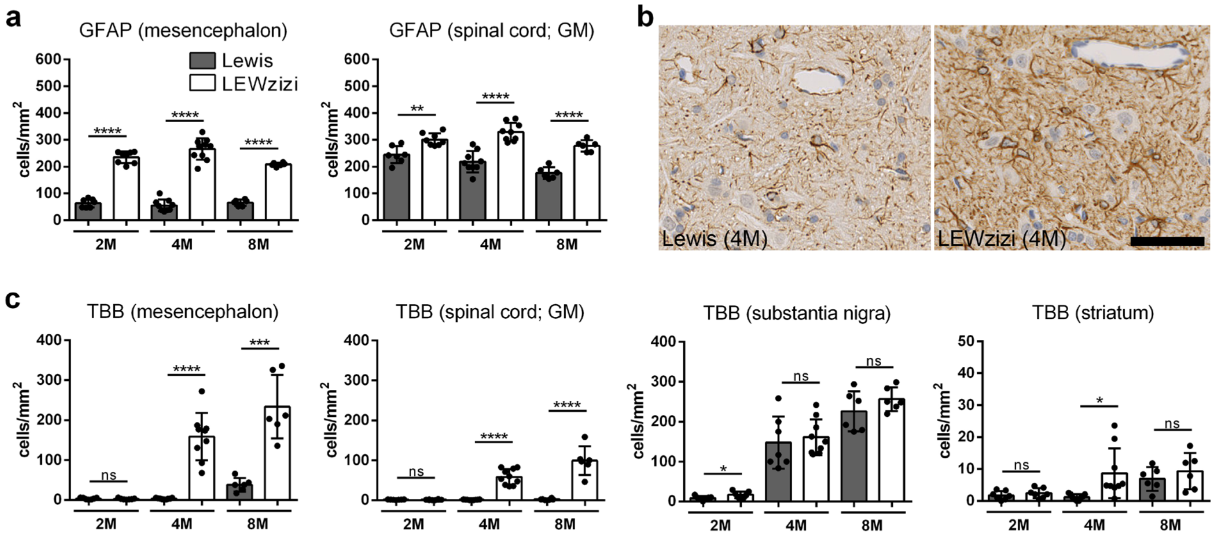

Supplementary Figure 3: Astrocytosis and iron accumulation in the LEWzizi CNS

(a) Quantification of immunohistochemical stainings for the astrocyte marker glial fibrillary acidic protein (GFAP) in the mesencephalon and lumbar spinal cord grey matter (GM) of 2-month-old (2M), 4-month-old (4M) and 8-month-old (8M) Lewis and LEWzizi rats.

(b) Immunohistochemistry for GFAP in 4M Lewis and LEWzizi lumbar spinal cord sections. Scale bar, 50 µm

(c) Quantification of DAB-enhanced Turnbull Blue (TBB) stainings for the detection of non-haem tissue iron. Only parenchymal cells were counted. Perivascular/meningeal cells and residual cells in the lumen of blood vessels were omitted from any analysis. Cells were quantified in the mesencephalon, lumbar spinal cord grey matter (GM), substantia nigra and striatum of 2M, 4M and 8M Lewis and LEWzizi rats.

(a, c) Bar graphs represent mean ± SD. Data points represent individual rats. Reported statistics result from unpaired, two-tailed Student's t-tests. *, p-value < 0.05; ***, p-value < 0.01; ***, p-value < 0.001; ****, p-value < 0.0001; ns, not significant

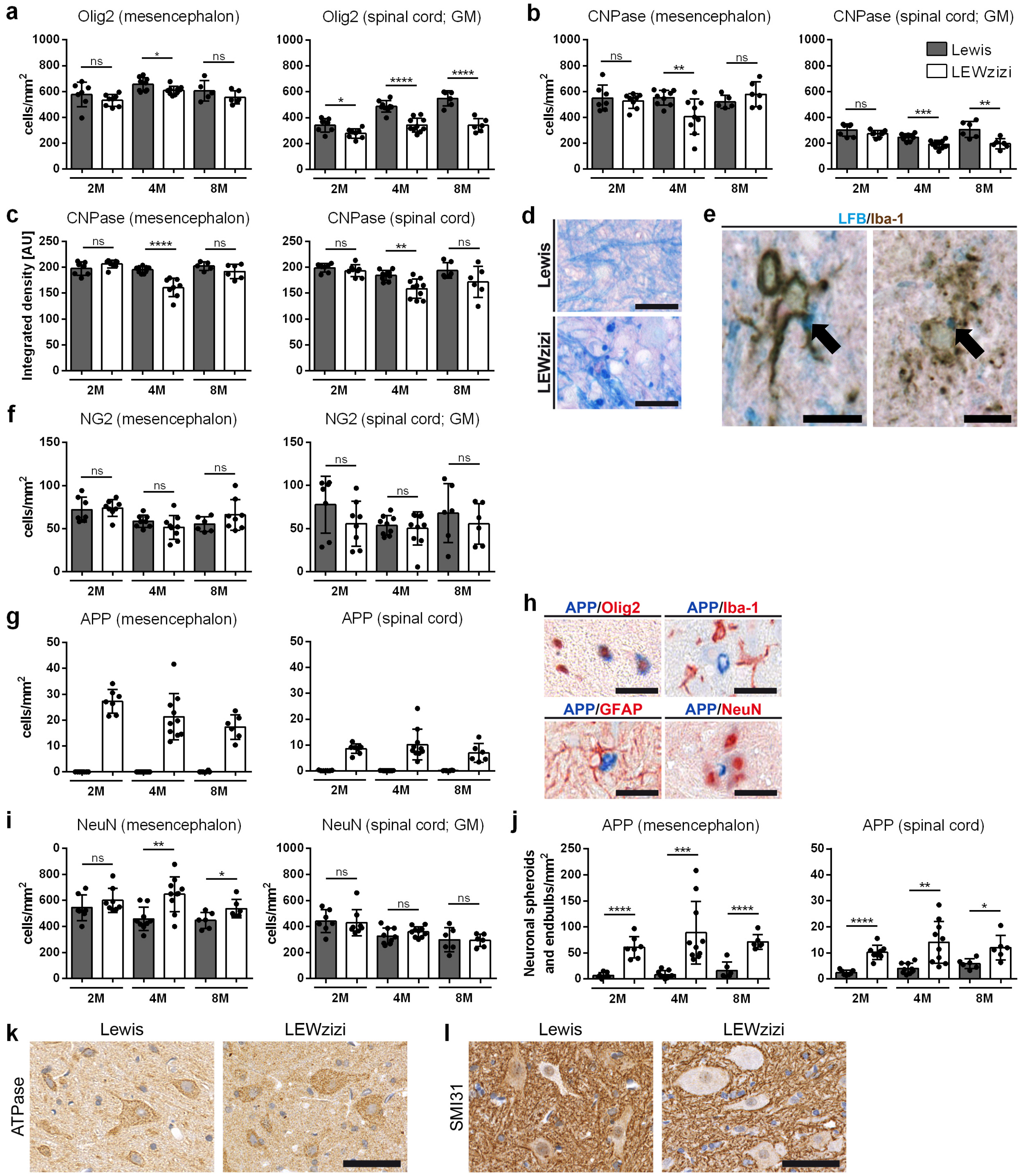

Supplementary Figure 4: Oligodendrocyte, myelin and axonal pathologies in the LEWzizi CNS

(a, b, f, g, i, j) Quantification of immunohistochemical stainings for Olig2 (a), CNPase (b), NG2 (f), APP (g, j) and NeuN (i). Positively stained parenchymal cells or axonal spheroids/endbulbs were quantified in the mesencephalon and spinal cord grey matter (GM) of 2-month-old (2M), 4-month-old (4M) and 8-month-old (8M) Lewis and LEWzizi rats. Bar graphs represent mean ± SD. Data points represent individual rats. Reported statistics result from unpaired, two-tailed Student's t-tests. *, p-value < 0.05; **, p-value < 0.01; ***, p-value < 0.001; ****, p-value < 0.0001; ns, not significant **(c)** Determination of myelin densities. Pictures were taken from the mesencephalon and spinal cord and subjected to densitometric analysis measuring the integrated density of CNPase stainings. Results are presented in arbitrary units (AU). **(d)** Luxol fast blue (LFB)-stained myelinated fibers and myelin adducts in the spinal cord GM of 8M rats. Scale bars, 20 µm **(e)** Double labelling of Iba-1 with LFB in the lumbar spinal cord GM of a 4M LEWzizi rat. Arrows point towards myelin adducts within microglia. Scale bars, 10 µm **(h)** Double labelling of APP with Iba-1 (microglia), Olig2 (oligodendrocytes), NeuN (neurons) and GFAP (astrocytes). Scale bars, 25 µm **(k)** Immunohistochemical staining of mitochondrial respiratory chain complex V (ATPase) showing the expected mitochondrial staining pattern in both Lewis and LEWzizi (both 4M) neurons of the lumbar spinal cord. In case of severe neuronal dysfunction, a pronounced cytoplasmic staining would have been detectable. Scale bar, 50 µm **(l)** Immunohistochemical staining of phosphorylated neurofilament H (SMI31) labelling thin and thick axons in both Lewis and LEWzizi (both 4M) lumbar spinal cord sections. In case of neuronal pathologies, a marked immunoreactivity in neuronal cell bodies would have been visible. Scale bar, 50 µm

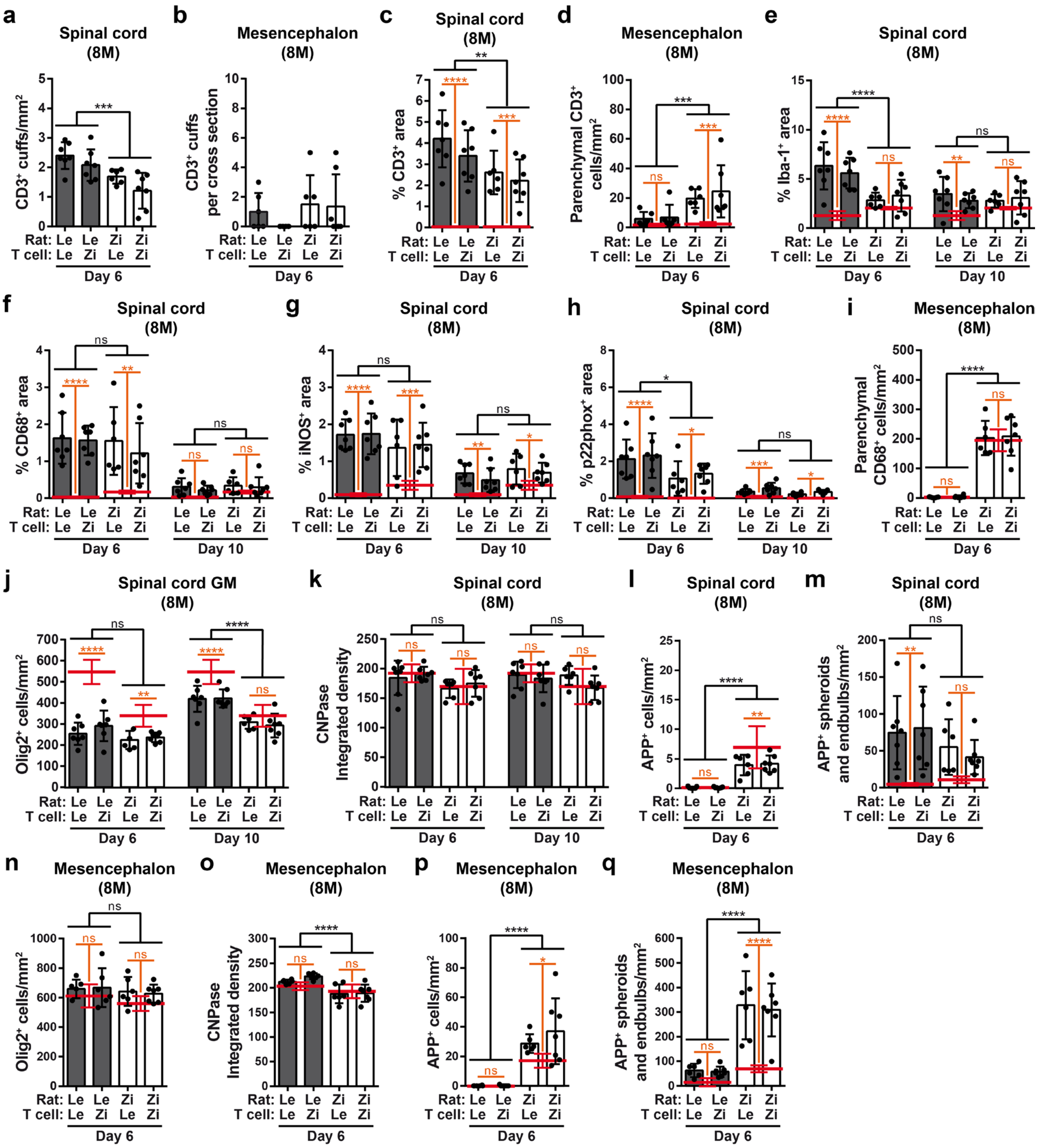

Supplementary Figure 5: Quantification of neuroinflammation, myelin pathology and neuronal damage in MBP-EAE rats

Tissues from 8-month-old (8M) EAE rats were harvested at the peak of disease (day 6) and during the recovery phase (day 10). If applicable, results for age-matched naïve rats are indicated by a red line for better comparability of up-/down-regulations during EAE. **(a, b)** Quantification of CD3$^+$ T cell-containing perivascular cuffs in the lumbar spinal cord (a) and mesencephalon (b). **(c, e-h)** Area fraction analysis of CD3 (c), Iba-1 (e), CD68 (f), iNOS (g) and p22phox (h) immunohistochemical stainings of lumbar spinal cord cross sections of 4-month-old (4M) Lewis and LEWzizi rats at the peak (day 6) and during the recovery phase (day 10) of EAE. The percentage of positively labelled area is depicted. **(d, i)** Quantification of CD3 (d) or CD68 (i) stainings of coronal brain sections. Only parenchymal cells were counted; perivascular/meningeal cells, residual cells in the lumen of blood vessels or cells in inflammatory cuffs were omitted from any analyses. **(j, n)** Quantification of Olig2 stainings in the spinal cord GM (j) and mesencephalon (n) **(k, o)** Myelin densities in the lumbar spinal cord (k) and mesencephalon (o) determined by densitometric analysis measuring the integrated density of CNPase stainings. Results are presented in arbitrary units. **(l, p)** Quantification of cells with a strong cytoplasmic APP staining in lumbar spinal cord cross sections (l) and the mesencephalon (p). **(m, q)** Quantification of APP-positive neuronal spheroids and endbulbs in the lumbar spinal cord (m) and mesencephalon (q).

Graphs represent mean ± SD. Data points represent individual rats. Red lines indicate the mean ± SD of age-matched naïve Lewis or LEWzizi controls. Experimental groups comprise 6-8 rats each. Statistics result from two-way ANOVAs (separate analyses for day 6 and day 10) reporting (i) differences between Lewis EAE rats and LEWzizi EAE rats by black bars and (ii) differences between naïve controls rats and EAE rats by orange bars. Data were pooled according to rat genotype and independent of T cell genotype. *, p-value < 0.05; **, p-value < 0.01; ***, p-value < 0.001; ****, p-value < 0.0001; ns, not significant

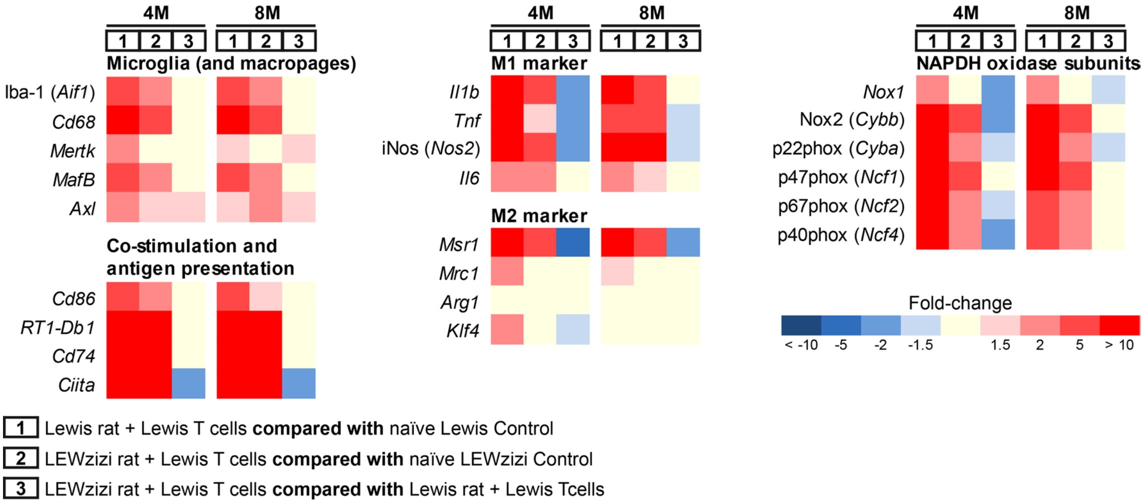

Supplementary Figure 6: Gene expression profiling in spinal cord tissue from EAE rats

Microglia- and inflammation-associated genes were analysed via TaqMan qPCR using lumbar spinal cord samples of 4-month-old (4M) and 8-month-old (8M) Lewis and LEWzizi rats each injected with Lewis T cells. Tissues were sampled at the peak of EAE and gene expression fold-changes of the EAE samples were calculated in comparison with either naïve age-matched Lewis and LEWzizi control tissues (columns 1 and 2, respectively) or between each other (column 3).

Supplementary References

1. Bauer J, Lassmann H (2016) Neuropathological Techniques to Investigate Central Nervous System Sections in Multiple Sclerosis. Methods Mol Biol 1304:211-229. doi:10.1007/7651_2014_151

2. Butovsky O, Jedrychowski MP, Moore CS, Cialic R, Lanser AJ, Gabriely G, Koeglsperger T, Dake B, Wu PM, Doykan CE, Fanek Z, Liu L, Chen Z, Rothstein JD, Ransohoff RM, Gygi SP, Antel JP, Weiner HL (2014) Identification of a unique TGF-beta-dependent molecular and functional signature in microglia. Nat Neurosci 17:131-143. doi:10.1038/nn.3599

3. Chiu IM, Morimoto ET, Goodarzi H, Liao JT, O'Keeffe S, Phatnani HP, Muratet M, Carroll MC, Levy S, Tavazoie S, Myers RM, Maniatis T (2013) A neurodegeneration-specific gene-expression signature of acutely isolated microglia from an amyotrophic lateral sclerosis mouse model. Cell Rep 4:385-401. doi:10.1016/j.celrep.2013.06.018

4. Gautier EL, Shay T, Miller J, Greter M, Jakubzick C, Ivanov S, Helft J, Chow A, Elpek KG, Gordonov S, Mazloom AR, Ma'ayan A, Chua WJ, Hansen TH, Turley SJ, Merad M, Randolph GJ (2012) Gene-expression profiles and transcriptional regulatory pathways that underlie the identity and diversity of mouse tissue macrophages. Nat Immunol 13:1118-1128. doi:10.1038/ni.2419

5. Hickman SE, Kingery ND, Ohsumi TK, Borowsky ML, Wang LC, Means TK, El Khoury J (2013) The microglial sensome revealed by direct RNA sequencing. Nat Neurosci 16:1896-1905. doi:10.1038/nn.3554

6. Keren-Shaul H, Spinrad A, Weiner A, Matcovitch-Natan O, Dvir-Szternfeld R, Ulland TK, David E, Baruch K, Lara-Astaiso D, Toth B, Itzkovitz S, Colonna M, Schwartz M, Amit I (2017) A Unique Microglia Type Associated with Restricting Development of Alzheimer's Disease. Cell 169:1276-1290 e1217. doi:10.1016/j.cell.2017.05.018

7. King G, Payne S, Walker F, Murray GI (1997) A highly sensitive detection method for immunohistochemistry using biotinylated tyramine. J Pathol 183:237-241

8. Krasemann S, Madore C, Cialic R, Baufeld C, Calcagno N, El Fatimy R, Beckers L, O'Loughlin E, Xu Y, Fanek Z, Greco DJ, Smith ST, Tweet G, Humulock Z, Zrzavy T, Conde-Sanroman P, Gacias M, Weng Z, Chen H, Tjon E, Mazaheri F, Hartmann K, Madi A, Ulrich JD, Glatzel M, Worthmann A, Heeren J, Budnik B, Lemere C, Ikezu T, Heppner FL, Litvak V, Holtzman DM, Lassmann H, Weiner HL, Ochando J, Haass C, Butovsky O (2017) The TREM2-APOE Pathway Drives the Transcriptional Phenotype of Dysfunctional Microglia in Neurodegenerative Diseases. Immunity 47:566-581 e569. doi:10.1016/j.immuni.2017.08.008

9. Zhang Y, Chen K, Sloan SA, Bennett ML, Scholze AR, O'Keeffe S, Phatnani HP, Guarnieri P, Caneda C, Ruderisch N, Deng S, Liddelow SA, Zhang C, Daneman R, Maniatis T, Barres BA, Wu JQ (2014) An RNA-sequencing transcriptome and splicing database of glia, neurons, and vascular cells of the cerebral cortex. J Neurosci 34:11929-11947. doi:10.1523/jneurosci.1860-14.2014
